# Reconstructing the Dynamics of HIV Evolution within Hosts from Serial Deep Sequence Data

**DOI:** 10.1371/journal.pcbi.1002753

**Published:** 2012-11-01

**Authors:** Art F. Y. Poon, Luke C. Swenson, Evelien M. Bunnik, Diana Edo-Matas, Hanneke Schuitemaker, Angélique B. van 't Wout, P. Richard Harrigan

**Affiliations:** 1 BC Centre for Excellence in HIV/AIDS, Vancouver, British Columbia, Canada; 2 Department of Experimental Immunology, Sanquin Research, Landsteiner Laboratory, Center for Infection and Immunity Amsterdam, Academic Medical Center, University of Amsterdam, Amsterdam, The Netherlands; 3 Department of Cell Biology and Neuroscience, University of California, Riverside, California, United States of America; 4 Crucell Holland BV, Leiden, The Netherlands; Imperial College London, United Kingdom

## Abstract

At the early stage of infection, human immunodeficiency virus (HIV)-1 predominantly uses the CCR5 coreceptor for host cell entry. The subsequent emergence of HIV variants that use the CXCR4 coreceptor in roughly half of all infections is associated with an accelerated decline of CD4+ T-cells and rate of progression to AIDS. The presence of a ‘fitness valley’ separating CCR5- and CXCR4-using genotypes is postulated to be a biological determinant of whether the HIV coreceptor switch occurs. Using phylogenetic methods to reconstruct the evolutionary dynamics of HIV within hosts enables us to discriminate between competing models of this process. We have developed a phylogenetic pipeline for the molecular clock analysis, ancestral reconstruction, and visualization of deep sequence data. These data were generated by next-generation sequencing of HIV RNA extracted from longitudinal serum samples (median 7 time points) from 8 untreated subjects with chronic HIV infections (Amsterdam Cohort Studies on HIV-1 infection and AIDS). We used the known dates of sampling to directly estimate rates of evolution and to map ancestral mutations to a reconstructed timeline in units of days. HIV coreceptor usage was predicted from reconstructed ancestral sequences using the geno2pheno algorithm. We determined that the first mutations contributing to CXCR4 use emerged about 16 (per subject range 4 to 30) months before the earliest predicted CXCR4-using ancestor, which preceded the first positive cell-based assay of CXCR4 usage by 10 (range 5 to 25) months. CXCR4 usage arose in multiple lineages within 5 of 8 subjects, and ancestral lineages following alternate mutational pathways before going extinct were common. We observed highly patient-specific distributions and time-scales of mutation accumulation, implying that the role of a fitness valley is contingent on the genotype of the transmitted variant.

## Author Summary

At the start of infection, human immunodeficiency virus (HIV) generally requires a specific protein receptor (CCR5) on the cell surface to bind and enter the cell. In roughly half of all HIV infections, the virus population eventually switches to using a different receptor (CXCR4). This ‘HIV coreceptor switch’ is associated with an accelerated rate of progression to AIDS. Although it is not known why this switch occurs in some infections and not others, it is thought to be shaped by constraints on how HIV can evolve from one mode to another. In this study, we test this hypothesis by reconstructing the evolutionary histories of HIV within 8 patients known to have undergone an HIV coreceptor switch. Each history is recreated from samples of HIV genetic sequences that were derived from repeated blood samples by next-generation sequencing, an emerging technology that is rapidly becoming an essential tool in the study of rapidly-evolving populations such as viruses or cancerous cells. Because we have samples from different points in time, we can use models of evolution to extrapolate back in time to the ancestors of each infection. Our analysis reveals patient-specific dynamics in HIV evolution that sheds new light on the determinants of the coreceptor switch.

## Introduction

Human immunodeficiency virus type 1 (HIV-1) enters into a host cell by binding the CD4 receptor and one of two HIV coreceptors, CCR5 or CXCR4. Most HIV-1 variants manifest preferential binding to one or the other coreceptor, a phenotype that is referred to as HIV coreceptor usage or tropism. HIV populations are predominantly CCR5-using at the start of infection and switch to being CXCR4-using in roughly 50% of HIV subtype B infections before progressing to AIDS [1], [2]; this proportion varies substantially among HIV subtypes with the highest reported in subtype D [3]. This HIV coreceptor switch is clinically significant because it is associated with accelerated deterioration of the CD4+ T-cell population and rate of progression to AIDS [1], [2]. In addition, a new class of antiretroviral drugs (HIV coreceptor antagonists) inhibit HIV infection by competitively binding the CCR5 coreceptor. A patient carrying detectable CXCR4-using variants is essentially not responsive to this class of drugs [4]. Despite its clinical significance, the biological determinants underlying the evolution of the HIV coreceptor switch remain poorly understood [5].

HIV coreceptor usage is a genetically complex phenotype. The primary genetic determinant is the third variable region (V3) of the HIV gp120 envelope glycoprotein comprising a disulfide-bonded loop that varies between 30 and 40 amino acids in length. The presence of basic residues at V3 reference positions 11 and 25 is strongly predictive of CXCR4 usage [6] but there are many exceptions to this rule. Although as few as one or two amino acid replacements in V3 may be sufficient to change coreceptor usage [7], the earliest detectable CXCR4-using viruses *in vivo* tend to carry additional compensatory mutations in V3 [8]. The effects of mutations in V3 can also be modulated by mutations within other regions of the HIV envelope glycoprotein [9]. Furthermore, the V3 region is targeted by both the cellular and humoral immune responses and undergoes extremely rapid host-specific adaptation [10], which may influence evolution of CXCR4 use. Consequently, CXCR4 use could potentially evolve through a series of intermediate genotypes (mutational pathways) that are unique to each individual.

**Figure 1:**
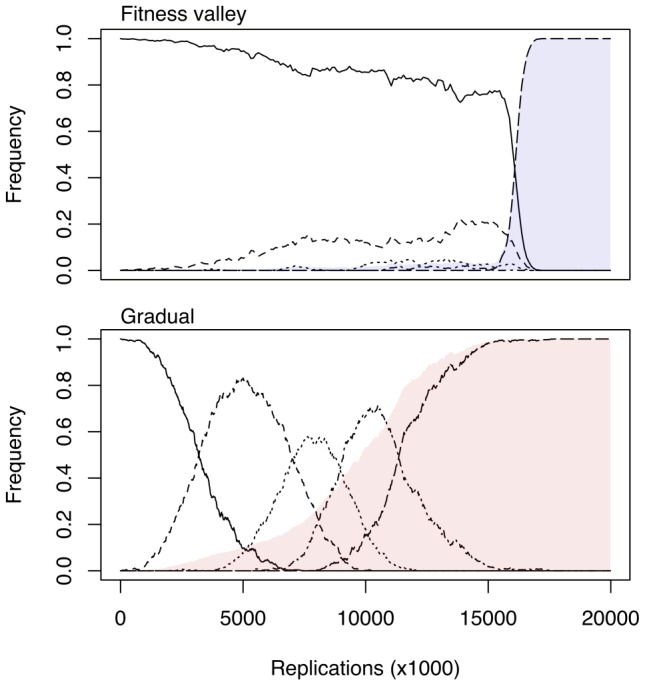
Simulated trajectories of genotype frequencies (solid and dashed lines) and population-level coreceptor usage phenotype (shaded regions) under the fitness valley and gradual models of HIV coreceptor usage evolution. Simulations were generated under a five-allele Moran model with mortality selection [42], effective population size 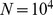, forward mutation rate of 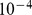 per replication, and fitness vectors of (1, 1.025, 1.05, 1.075, 1.1) and (1, 0.999, 0.999, 0.999, 1.1) corresponding to gradual and valley landscapes, respectively. Note that the relatively rapid and complete fixation of the fifth variant is partly due to the model assumption of no back mutation, and is not consistent with the observation that CXCR4-using variants tend to remain a minority species in HIV infections.

The nature of the mutational pathway to evolving CXCR4 usage is postulated to be a significant determinant of the limited incidence of the HIV coreceptor switch [5]. If CCR5- and CXCR4-using genotypes are separated by intermediate genotypes of reduced fitness, then the traversal of this ‘fitness valley’ is a chance event that might never occur over the course of an HIV infection. Negative selection prevents intermediate genotypes from reaching substantial frequencies in the population. As a result, any lineage must rapidly accumulate multiple mutations to reach the CXCR4 genotype before going extinct; this process is known as ‘stochastic tunnelling’ [11]. In contrast, if the pathway passes through intermediates of progressively greater fitness, then CXCR4 usage evolves by the gradual and deterministic accumulation of mutations that would unfold at a similar rate in all individuals (Figure 1). Reconstructing the evolutionary history of CXCR4 usage within individuals would lend important insight into which model better explains the evolution of HIV coreceptor tropism and hence the genetic determinants of HIV pathogenesis. Specifically, we want to determine whether the dynamics of the evolution of HIV coreceptor use in these subjects was consistent with a gradual (immediate and slow) or fitness valley model (delayed and rapid; Figure 1).

Recently, an exceptional set of HIV genetic sequence data was collected for the purpose of identifying putative evolutionary intermediates in eight chronically-infected subjects from the Amsterdam Cohort Studies on HIV infection and AIDS (ACS) whose virus populations had undergone an HIV coreceptor switch [12]. These data were generated by ‘deep sequencing’, an application of next-generation sequencing technology for large-scale automated clonal sequencing of individual nucleic acids along a fixed interval [13]. By generating thousands of clonal sequences per amplicon, deep sequencing can provide a detailed sample of the genetic variation in a virus population. Accordingly, it has been used with success to reliably detect drug resistant HIV variants at frequencies as low as 2% in the population [14], [15]. Based on deep sequence data generated from serial samples of HIV at three month intervals, Bunnik and colleagues [12] were able to observe HIV sequence variants that were intermediate of the predominant CCR5- and CXCR4-using variants in these samples as determined by a minimum spanning tree, the shortest acyclic graph connecting all sequences in the sample where distance was quantified by the number of nucleotide differences (Hamming distance). While a minimum spanning tree can provide a useful visual representation of the genetic diversity in a sample, it should not be construed as representing the evolutionary history of the sample. First, a minimum spanning tree can only traverse the set of observed sequences. It does not attempt to reconstruct the ancestors from which the observed sequences descended. This limitation is problematic if a significant amount of the evolution of HIV coreceptor use takes place prior to the time of the first sample, or between subsequent samples. Second, a minimum spanning tree does not explicitly incorporate a time dimension. Connections in the graph are drawn between sequences irrespective of time of sampling, which can make it exceedingly difficult to interpret the minimum spanning tree with respect to time. For instance, it is difficult to assess from a minimum spanning tree the rate at which evolution has unfolded, which is a prerequisite to differentiate between the gradual and fitness valley models of the HIV coreceptor switch.

In this study, we apply a phylogenetic modelling framework to these data with the direct objective of reconstructing the evolutionary history of HIV coreceptor use within subjects over time. Like a minimum spanning tree, a phylogeny is an acyclic graph that connects observed sequences. Generally speaking, however, a phylogeny is a bifurcating tree that relates 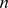 observed sequences at its tips by incorporating 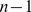 latent nodes that represent a hierarchy of the most recent common ancestors of the sample (Supporting Figure S1). Put another way, a phylogeny directly models the evolutionary relationships among observed sequences. It can therefore be used as a template to fit models of sequence evolution that are typically implemented as a continuous-time Markov model [16]. Furthermore, efficient algorithms have been developed for extracting maximum likelihood reconstructions of ancestral character states from the combination of the phylogeny and a model of evolution [17].

The use of phylogenetic methods enables us to take advantage of the known dates of sampling in these data to directly estimate the rates of HIV evolution along lineages within each subject over time. This practice is known as reconstructing a phylogeny with ‘dated tips’ [18], which enables one to measure the heights of ancestral nodes in the tree in units of real time (such as days or years). By reconstructing ancestral sequences at these nodes, we can deduce that one or more mutations have occurred along the branch between two nodes from differences in the corresponding sequences [19] and thereby estimate when each mutation first arose in the population. This is crucial information for quantifying the dynamics of virus evolution over time. Here we show how the phylogenetic analysis of the serial deep sequence data from the ACS cohort can reconstruct the dynamics of HIV coreceptor usage evolution in each population, ranging from the estimated start of infection to the last date of sampling.

## Methods

### Ethics statement

The Amsterdam Cohort Studies on HIV-1 infection and AIDS (ACS) have been conducted in accordance with the ethical principles set out in the Declaration of Helsinki, and written informed consent was obtained prior to data and material collection. The study was approved by the Academic Medical Center institutional medical ethics committee.

### Data collection

The individuals included in our present study were men who have sex with men participating in the ACS who were seropositive and asymptomatic at enrolment into the cohort between 1988 and 1994 [20]. Blood samples were obtained at approximately 3 month intervals from 8 participants who had at least three negative MT-2 assays in the 12 months prior to their first positive MT-2 assay result. MT-2 is a human lymphoblastoid cell line that is highly susceptible to infection by CXCR4-using HIV, which is manifested by the formation of multinucleate cells (syncytia). We will refer to the time of the first positive MT-2 syncytium induction (SI) assay as 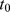 (‘time zero’). Viral loads associated with these samples were reported in a previous study, where we have retained the anonymized identifiers for study participants (DS1 to DS8) [12]. Over the course of follow-up, all individuals in the present study were eventually verified as having a CXCR4-using infection using an *in vitro* recombinant virus assay (Trofile) and by deep sequencing [12].

Virus sequences were classified by genotype (*env* V3 region) as CXCR4-using by the geno2pheno (g2p) algorithm [21]. The g2p algorithm is a support vector machine-based classifier trained on a database of predominantly clonal V3 sequences labelled with HIV coreceptor tropism as determined by cell-based assays. It yields a predictive score that is conventionally mapped to an empirical false positive rate (FPR) distribution for interpretation. Based on previous studies [4], [22], we use an FPR cutoff of 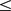
3.5 to classify sequences as CXCR4-using. At this cutoff, the algorithm predicts that 3.5% of CCR5-using sequences would be misclassified as CXCR4-using.

In this study, the range of samples subjected to deep sequencing has been expanded from the range reported in [12] (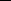
12 to 0 months relative to 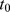) to encompass up to 24 months prior and up to 6 months subsequent to 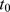 (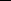
24 to 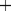
6 months). Using a NucliSENS easyMAG (bioMérieux), HIV RNA was extracted from 500 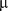
L from previously frozen serum samples and eluted in 60 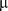
L of buffer. Three aliquots of 4 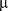
L eluate each (12 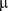
L total) were transferred to triplicate RT-PCR amplification reactions using the SuperScript II OneStep RT-PCR system with Platinum *Taq* High Fidelity enzyme (Invitrogen). These amplicons in triplicate were independently amplified in second-round PCR reactions using the Expand High Fidelity PCR system (Roche) with primers that were specific to the interval of the HIV-1 genome surrounding the *env* V3 region (HXB2 reference nucleotides (7085–7372) [12] and which incorporated unique 10bp sequence ‘tags’ (also known as barcodes) for multiplexed pyrosequencing [23]. The triplicate second-round amplicons were pooled in equal quantities for deep sequencing on Roche/454 GS-FLX or GS Junior platforms as previously described in [4].

The diluting effects of this experimental protocol will inevitably reduce the number of nucleic acids in the sample represented by copies available for clonal sequencing by the 454 platform. This dilution could have a detrimental effect on phylogenetic reconstruction. In the extreme case, if all templates being sequenced were descended from a single copy of HIV RNA in the blood specimen, then the phylogeny would only reflect genetic divergence due to sequencing error. Suppose that there were 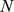 copies of HIV RNA in 1 mL of serum. This number was immediately halved as 500 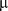
L was used for extraction. The number of nucleic acids that entered the RT-PCR step was determined in part by the efficiency of viral RNA extraction by the NucliSENS easyMAG, which we estimated to be about 44% (Supporting Text S1). Hence, we expect that about 0.22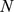 nucleic acids would have been present in the extraction eluate. Using 12 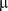
L from 60 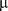
L of eluate for RT-PCR would have further reduced the number of nucleic acids to 0.044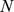. Subsequent dilution due to variation in amplification rates among initial nucleic acids would have been ameliorated by carrying out RT-PCR in triplicate. Therefore, we estimate an approximate 20-fold dilution factor due to sample processing. Viral loads reported from these chronic untreated subjects were generally high with a median of 17,000 HIV RNA copies/mL [12]. Consequently, we estimate templates in the amplicon available for sequencing would have been derived from about 850 HIV RNA copies in the original specimen. We randomly subsampled 50 sequences per time point for phylogenetic reconstruction (see below). Using a Poisson approximation validated by simulation, we estimated that the median per-molecule probability of resampling (appearing twice or more in a sample of 50 sequences) was about 0.17%.

### Data processing and sequence alignment

The raw sequence output (‘reads’) generated by Roche/454 GS-FLX or GS Junior platforms was processed by a Ruby script that sorted reads by region, tag and primer; trimmed low quality bases from the start and end of reads (according to quality scores reported by Roche/454 GS software version 1.1); and temporarily collapsed identical reads into unique sequence entries annotated by read count. We retained the entire read lengths (averaging about 250 bp) for phylogenetic analysis; in a previous study, the reads had been clipped to the V3 region (105 bp) [12]. The resulting files were processed using a custom sequence alignment module implemented in *HyPhy* [24]. This module was designed to compensate for the high rate of insertions and deletions (indels) introduced by pyrosequencing-based platforms by aligning all three reading frames of each sequence against a reference protein sequence (HXB2 gp120 residues 278–375). We assumed that true HIV coding sequences maintained a single reading frame along their entire length, such that any frame-shifts represented nucleotide indel errors induced by pyrosequencing. This algorithm is described in Supporting Text S2.

The resulting sequences were grouped by patient, re-expanded by read counts, and annotated by sample dates in units of days since January 1, 1990. For each patient, 50 sequences were randomly subsampled without replacement from every sampling time point (averaging 3642 reads per sample) for a median of 350 sequences total. This step was necessary to reduce the number of all possible trees to a level at which a Markov chain Monte Carlo (MCMC) sample could converge to the posterior distribution within a feasible amount of time (see below). A multiple sequence alignment was generated for each set of sequences using MUSCLE version 3.8.31 [25] with diagonal optimization and a single iteration, and refined manually using the alignment viewer Se-Al (Andrew Rambaut, http://tree.bio.ed.ac.uk/software/seal/). The final sequence alignments, annotated by specimen and collection date, have been deposited in the public Genbank database (accession numbers JX561243-JX564138). Additionally, the unprocessed short read data have been deposited to the European Nucleotide Archive (study accession number ERP001795, run accession numbers ERR169842-ERR169899).

### Tree sampling

We used BEAST [26] to reconstruct dated-tips phylogenies from these data. BEAST uses a Bayesian MCMC procedure to sample trees from the posterior distribution given the sequence data and a prior distribution that is usually set to the coalescent model. Each alignment representing serial samples of HIV sequences from a given patient was converted into a BEAST XML format using a custom Python script. These conversions were based on a template XML file with the following settings: Tamura-Nei [27] nucleotide substitution model with rate variation across sites modelled by a discretized gamma distribution with 4 rate categories, and with substitution rates and bias parameters unlinked between codon positions 1 and 2 and position 3 [28]; an uncorrelated lognormal molecular clock; a Bayesian skyline model with 3 population size classes; and a chain length of 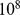 steps with chain states written to log files at regular intervals of 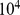 steps. These settings were chosen on the basis of preliminary runs on these data and previous experience [29]. Chains were seeded with a random coalescent tree. All chain samples were executed in parallel on a Beowulf cluster using BEAST version 1.6.1 with a native-compiled likelihood core. We ran two replicate chains for each XML file to assess convergence. We assessed the effect of subsampling 50 sequences per time point by running additional chain samples on a second set of randomly subsampled sequence alignments and observed no qualitative differences in results based on phylogenetic or ancestral sequence reconstruction (see Supporting Figure S2).

Convergence in chain samples was assessed using Gelman and Rubin's convergence diagnostic as implemented in the R package *coda* [30]. This diagnostic reports a potential scale reduction factor (PRSF) that is a conservative estimate of the ratio between the pooled variance across replicate chains to the variance within chains [31]. Values of PRSF that are substantially greater than 1 indicate a lack of convergence such that the chain samples are still influenced by their initial values, such as when a chain becomes trapped on a local optimum. If the upper confidence interval in estimation of PRSF exceeded 1.25 for replicate chains, then we ran additional chain samples from the same BEAST XML file for a longer number of steps and re-evaluated their PRSF. Plots of posterior traces from replicate chain samples are provided as Supporting Figure S3. Newick string representations of 100 trees were extracted from the log files at regular intervals following a burn-in period of 20% (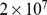 steps by default).

### Ancestral reconstruction

A Muse-Gaut [32] codon substitution model crossed with a general time-reversible model of nucleotide substitution [33] was fit to every tree in the thinned sample for a given sequence alignment using maximum likelihood heuristics implemented in HyPhy [24]. Branch lengths in each tree, which were expressed in units of days, were constrained to scale by a global factor when estimating the expected number of substitutions per codon site. Constraining the codon tree to remain congruent to the input tree not only speeds up computation but also preserves the relative differences in branch lengths inferred under a molecular clock. For a given fitted codon model and tree sample, ancestral sequences were generated by sampling 100 character states from the posterior distributions reconstructed at every node of the tree [34]. This approach is similar to the hierarchical Bayes approach to ancestral reconstruction [35] that integrates over the uncertainty in estimation of tree parameters (such as tree topology and branch lengths). Because codon substitution models were originally developed to compare non-synonymous and synonymous rates of substitution, stop codons are not permitted. As a result, it was necessary to censor any stop codons in the sequence alignments with gap characters, which are conventionally handled as fully ambiguous codons.

Codon substitution models generally do not model insertions or deletions (indels), and gaps are typically handled as missing data that can be resolved into any codon with equal probability. As a result, insertion polymorphisms in the observed sequences would be propagated to all ancestors when reconstructing sequences from a fitted codon model. This approach is not adequate for our purposes because HIV populations within hosts commonly contain legitimate indel polymorphisms in the *env* gene, and sequence length variation in the HIV-1 *env* V3 region can influence the HIV coreceptor tropism phenotype. Indeed, predictive models of HIV coreceptor tropism often incorporate the presence or absence of indels in V3 relative to a reference sequence [21]. Accordingly, we implemented a method to reconstruct indel character states in the ancestral codon sequences. First, we identified and encoded indel polymorphisms in the observed sequences as integer values using an algorithm implemented in Python (see Supporting Text S3).

In brief, an indel polymorphism was defined as a contiguous interval in the alignment containing one or more codon gaps. This polymorphism may be comprised of two or more character states corresponding to the respective lengths and location of each codon insertion or deletion within the gapped interval. We encoded these indels by integer values in decreasing order of prevalence, such that 0 represented the most common character state. Next, we fit a model of indel evolution to the resulting alignment of integer-valued sequence encodings. The evolution of indels can be modelled as a finite-state continuous-time Markov process akin to those used to model the evolution of nucleotide and amino acid sequences [36]. Because the maximum number of character states in any indel polymorphism did not exceed 3 in these data, we used the following instantaneous rate matrix:
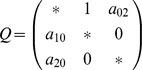
which assumes that states (1) and (2) are derived from (0), the most prevalent state, and that there are no transitions directly between (1) and (2). Rates are scaled arbitrarily to the rate of transition from state (0) to state (1). When indel rates were assumed to be reversible (equal rates of insertion and deletion affecting the same codons), we applied the constraints 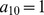 and 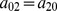; otherwise, indel evolution was non-reversible. Character frequencies were computed dynamically by setting all values in the right-most column of 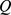 to 1 and extracting the last row of the matrix inverse. Both reversible and non-reversible models were fit using maximum likelihood heuristics in *HyPhy* to each sampled tree with branch lengths constrained to scale by a global factor to preserve the molecular clock characteristics of the tree as was applied to fitting the codon model (see above). Since the reversible model is a special case of the non-reversible model, we calculated the likelihood ratio test statistic (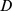) to select between the fitted models. Computing 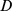 across replicate ancestral reconstructions, the reversible model was rejected only for subject DS2 (mean and interquartile range, 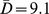 [7.1, 11.1], 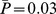 [0.004, 0.28]). We proceeded with the ancestral reconstruction of indel polymorphisms using the respective best models for each subject data set.

For each tree, 100 ancestral indel character states were sampled from the resulting posterior distributions at all internal nodes of the tree. These reconstructions were applied to the ancestral codon sequence reconstructions by overwriting nucleotides with gap characters according to the indel reconstructions. In total, we generated 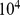 sets of ancestral reconstructions for each patient (100 trees
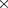
100 replicate samples). HIV coreceptor tropism predictions were generated for all ancestral sequences using the g2p algorithm and time-stamped by their heights in the trees, which we measured in units of days since an arbitrary date in the past.

### Visualization

For every tree in the sample, we tallied the relative frequencies of every clade (the subset of tips that descend from a given ancestral node) and then calculated the product of these frequencies for clades represented in each tree. The tree that maximized this product was taken as the most representative point estimate for the sample (the maximum credibility tree) [26]. For each replicate ancestral reconstruction, we mapped only the mutations in the V3 region that were predicted to increase the probability of CXCR4 usage (according to the g2p algorithm) to branches in the maximum credibility tree. We recorded the relative frequencies of these branches and identified the replicate that maximized the sum of these frequencies. In this context, we used the sum rather than the product to avoid penalizing replicates for mapping mutations to greater numbers of branches. We refer to the replicate that maximized this sum as the maximum ancestral reconstruction credible set (MARCS).

Each maximum credibility tree and corresponding MARCS was rendered in PostScript to visualize trends in the reconstructed evolution of HIV within patients. Consider 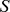 be the set of all ancestral nodes corresponding to the ends of branches to which one or more mutations in the MARCS was mapped. 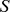 always included the root of the tree. Every node in 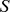 was plotted with its 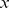 position representing its location on the timeline of the infection (ranging from the MRCA on the left to the last sampling time on the right), and its 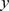 position representing the midpoint of the vertical positions of all its descendant extant sequences in the maximum credibility tree. For every node in 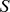, a line was drawn back to its most recent ancestor in 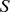. Consequently, any intervening branches that did not contain any mutations contributing to the evolution of CXCR4 usage were collapsed in this visualization. Each branch between members of 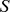 was rendered in a colour representing the FPR prediction from the g2p algorithm for the ancestral sequence reconstructed at the right-most (most recent) node. In addition, each branch was labelled with all reconstructed mutations contributing to CXCR4 usage that mapped to this branch. These procedures were automated using a HyPhy/Python pipeline.

Because these visualization strategies focused only on ancestral lineages accumulating mutations towards CXCR4 usage, we also visualized the overall diversification of HIV within hosts over time with respect to coreceptor usage predictions using 2-dimensional histograms. Across all 100 ancestral reconstructions on the maximum credibility tree, ancestral sequences were binned with respect to their predicted FPR value and time. The density of ancestral sequences in each bin, normalized for each time interval, was represented by degree of opacity for the bin's coloration in the 2-dimensional histogram. This visualization procedure was implemented in R using a modification of the *hist2d* function in the *gplots* package.

The entire workflow from raw sequence data to visualization is presented as a flowchart in Supporting Figure S4 and scripts used in our analysis are available at http://hyphy.org/wiki/Emerge.

## Results

### Molecular clock analysis

**Figure 2:**
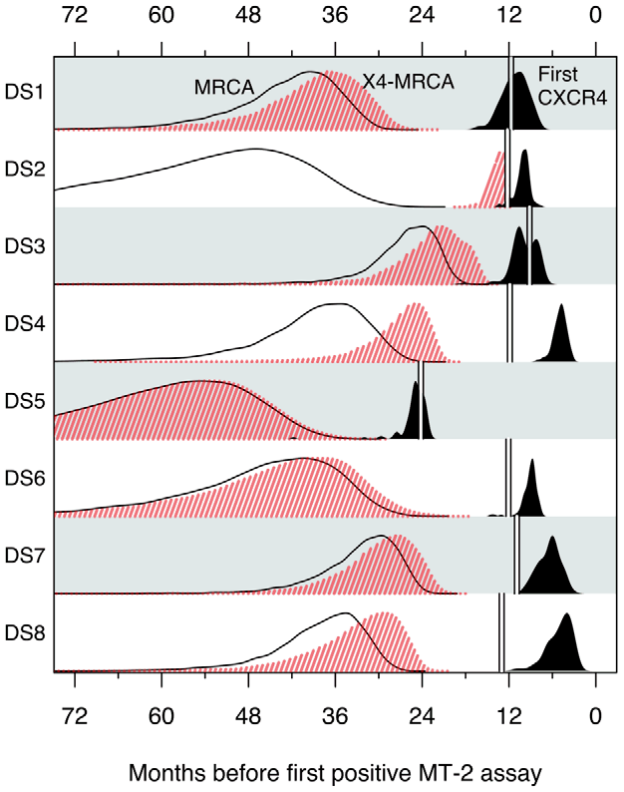
Kernel density estimates of the distribution of 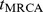 (solid line), 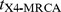 (red shaded region) and the time of the earliest CXCR4-using ancestor (defined at FPR
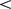
3.5; solid region) based on molecular clock analyses of HIV sequence variation from each subject. Double vertical lines indicate the time of the earliest sample per patient.

**Figure 3:**
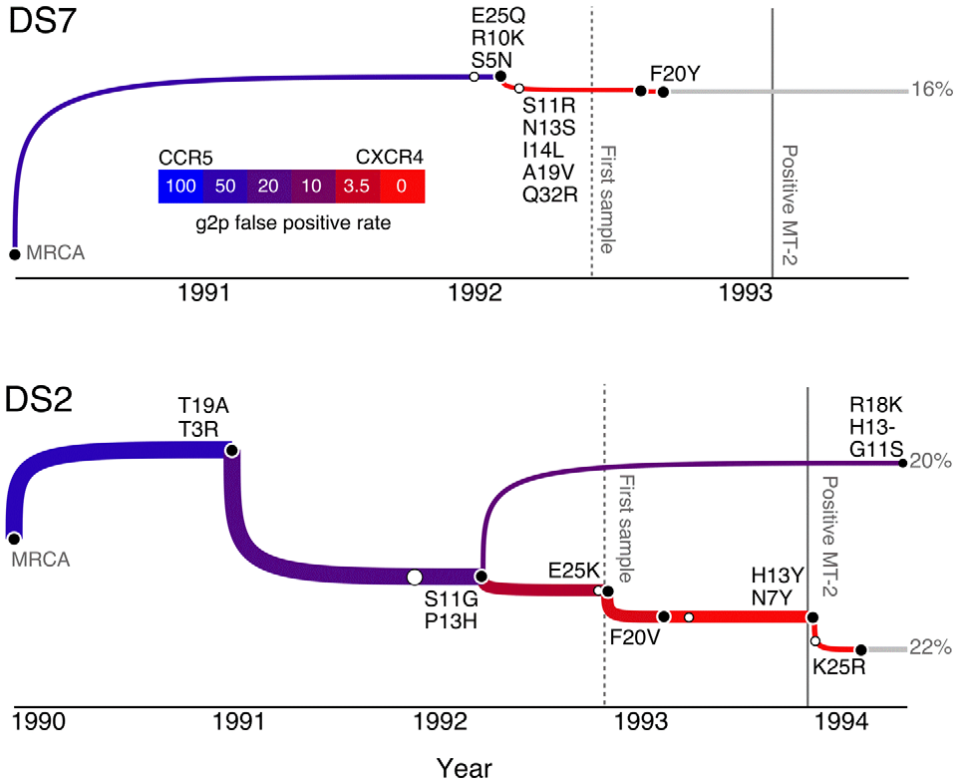
Excerpts from the maximum credibility trees for HIV evolution within subjects DS2 and DS7 with reconstructed mutations mapped to individual branches (labels comprising the ancestral residue, position in the V3 loop, and the derived residue). These excerpts emphasize the lineages that attained a CXCR4-using ancestral genotype (FPR
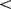
3.5). Branches are coloured with respect to the predicted FPR value (see legend inset). Vertical lines indicate the times of the first serum sample (dashed) and first positive MT-2 assay (solid), respectively. Open circles indicate the start of the branch carrying mutations promoting CXCR4 usage, which otherwise cannot be distinguished because other branches that do not carry such mutations have been collapsed. Percentiles indicate the fraction of the most recent sample that descend from the corresponding lineage.

The root of a phylogenetic tree estimates the most recent common ancestor (MRCA) of all individuals represented at the tips of the tree. Because the time of sampling is known for every tip in the tree, we can use the time elapsed between samples to directly estimate the rate of evolution (molecular clock) and extrapolate back to timing branching events deeper in the tree. Since HIV tends to undergo a severe population bottleneck at transmission, the time to the MRCA (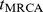) can provide a reasonable estimate of the time of HIV infection [29]. The median estimates of 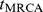 for each subject ranged from 25.7 to 59.6 months prior to the time of the first positive MT-2 assay (herein referred to as 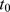) with an average of 41.3 months (Figure 2). Since the sample population consisted of longitudinal samples from infections undergoing an HIV coreceptor switch, initially determined by a transition from NSI (non-syncytium inducing) to SI phenotypes, we also extracted the times to the MRCA of all observed sequences that were predicted to be CXCR4-using according to the g2p algorithm with a false positive rate (FPR) cutoff of 3.5. This ancestor will be referred to as the X4-MRCA. Generally, estimates of times to the X4-MRCAs (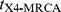) were similar to the corresponding estimates of 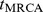 , indicating that CXCR4-using variants had emerged independently in multiple branches of the tree that did not converge until the MRCA (Figure 2). In one extreme exception, the 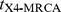 was estimated in subject DS2 to be about 40 months after the median 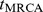 estimate and about 13.5 months prior to the 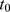 (Figure 2). In this case, mutations contributing to CXCR4 usage were reconstructed along one lineage only (see next section; Figure 3).

We used the coefficient of variation parameter (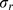) from the uncorrelated lognormal clock model to assess whether rates of evolution varied throughout each tree. A posterior distribution of 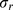 with a mode of zero indicates that one cannot reject a strict molecular clock model given the data [37]. For all subjects, the modes of the posterior distributions of 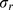 were substantially above zero, indicating significant variation in rates of evolution over time. Median clock rates among subjects ranged from 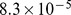 (DS2) to 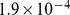 (DS3) mutations per nucleotide site per day. Variation in mean clock rate estimates among subjects was statistically significant (one-way analysis of variance, 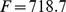, 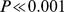).

### Emergence of CXCR4-using variants

Because the X4-MRCA was not necessarily itself a CXCR4-using variant, we reconstructed ancestral sequences throughout the phylogeny to determine the earliest predicted CXCR4-using ancestor for each subject. This was accomplished by sampling ancestral sequences from the posterior distributions reconstructed under combined models of codon and indel evolution at all internal nodes of each sampled tree. The times of the earliest ancestors predicted to be CXCR4-using by the g2p algorithm (FPR
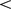
3.5) ranged from 4.6 to 24.7 months before 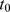 with a mean of 10.1 months (Figure 2). In other words, predicted ancestral CXCR4-using variants were on average present within a patient nearly one year prior to the first positive MT-2 assay. The time difference between the median estimate of 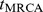 and the earliest CXCR4-using ancestor ranged from 15.6 to 43.5 months with a mean of 31.4 months (Figure 2). Because none of the participants received antiretroviral therapy over the course of the study [12], this result can be interpreted as estimating the expected waiting time for the first CXCR4-using variants to emerge as a product of the accumulation of genetic variation in an HIV infection in the absence of antiretroviral selection. These molecular clock results indicated that the evolution of CXCR4 usage could have unfolded over a period of one to several years within each subject. We carried out a detailed phylogenetic reconstruction of ancestral intermediates to characterize the dynamics of HIV coreceptor usage evolution over time.

### Pathways of evolving CXCR4 use

The evolution of CXCR4 use may require one to several amino acid replacements in V3, the primary genetic determinant of HIV coreceptor tropism. Because we have reconstructed the sequences for every ancestral node in the sampled trees, it is possible to map specific mutations to the branches of any given tree based on discordances in the reconstructed sequences on either end of a branch. To facilitate interpretation, we generated tree visualizations for only the maximum ancestral reconstruction credibility set (MARCS) on the maximum credibility tree. Figure 3 displays only the single lineages in the maximum credibility trees for subjects DS2 and DS7 that accumulated mutations culminating in a predicted FPR value below 3.5. The complete maximum credibility trees for all subjects are provided as Supporting Figure S5. In summary, whether the dynamics of HIV evolution was consistent with either fitness valley or gradual models depended on which subject was being evaluated. Subject DS2 provides an unambiguous example of the gradual accumulation of mutations contributing to CXCR4 use in a succession of intermediate genotypes over a period of roughly 3 to 4 years, culminating in the mutation K25R to yield a genotype with a predicted FPR of 0.6 under the g2p algorithm (Figure 3). In the final sample from DS2, 22% of the observed sequences were inferred to be descended from this ancestor. The first mutations to emerge (T19A and T3R) were mapped to a branch descending directly from the MRCA. Hence, the emergence of HIV coreceptor usage intermediates began soon after infection as predicted by the gradual model. On the other hand, multiple mutations occurred within a comparably narrow time interval of roughly half a year in subject DS7 (Figure 3) and only after a delay of nearly two years after 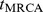 , which was consistent with the fitness valley model. For the remaining subjects, the emergence of mutations promoting CXCR4 use was generally more consistent with the gradual evolution observed for subject DS2 (Figure S5). We explored this trend in depth using additional visualization schemes reported below. In all cases, a substantial portion of HIV coreceptor evolution was mapped to ancestral lineages preceding the first sample, underscoring the importance of phylogenetic reconstruction.

An intriguing feature of the maximum credibility tree maps is that predicted CXCR4-using ancestors emerged in more than one lineage in 5 of 8 subjects (Supporting Figure S3). The largest number of lineages attaining a CXCR4-using genotype was 5 in subject DS6, although these lineages were related by a common ancestor preceding these endpoints by only about a year (Figure S5). We also observed evidence of parallel evolution along 3 lineages within DS8 that accumulated the mutations Q10R, S11G, H13R, D25K and the insertion −22A (Figure S5). In addition, the maximum credibility trees featured many lineages that accumulated a different set of mutations and failed to attain a CXCR4-using genotype before going extinct. For example, the main lineage leading to a CXCR4-using ancestor in subject DS1 accumulated the mutations L20M, K10R, G24E, Q25R and I27V with descendants comprising 44% of the last sample. A second lineage accumulated L20F, I27V, N29D and a deletion at Q25 to reach a predicted FPR of 5.7, leaving descendants comprising 22% of an earlier sample before going extinct (Figure S5). Consequently, lineage-specific dynamics of HIV coreceptor usage evolution may become obscured at the population level by other lineages following divergent mutational pathways.

**Figure 4:**
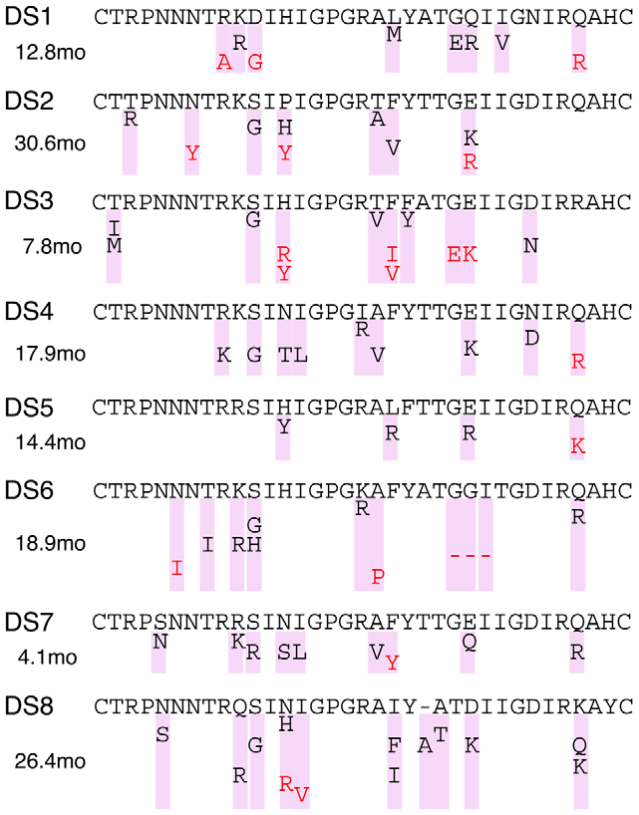
Mutations within the V3 loop comprising the predominant pathway for each subject, stratified by time of emergence. The MARCS V3 sequence reconstructed at the MRCA of the maximum credibility tree is shown at the top of each plot. Residues highlighted in red correspond to mutations that arose in a CXCR4-using background (FPR
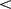
3.5). The duration of HIV coreceptor evolution from 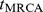 to the first CXCR4-using ancestor is indicated in months alongside each plot (see text).

To compare the locations of reconstructed mutations in the V3 region among subjects, we annotated the V3 amino acid sequence reconstructed at the root of the maximum credibility tree with the predominant mutational pathway, which was defined as comprising all mutations contributing to CXCR4 use that were mapped to the lineage with the largest proportion of descendants in the most recent sample (Figure 4). This visualization makes it clear that the predominant mutational pathways followed by each HIV population are highly divergent among subjects. The total numbers of mutations contributing to a CXCR4-usage prediction ranged from 4 to 13 (median 9) mutations per pathway. Mutations occurred most often at V3 loop positions 11 and 25 (in the predominant pathways of 7 and 8 subjects, respectively), followed by positions 13, 19, 20 and 32 (Figure 4). Insertions and deletions played a significant role in the evolution of CXCR4 usage. Specifically, the predominant pathways in DS6 and DS8 included a deletion at V3 positions 24–26 and an insertion at position 22 in DS8 serum, respectively, which we were able to reconstruct by incorporating a model of indel evolution into the ancestral reconstruction procedure (see Methods).

For all subjects, we quantified the time scale of CXCR4 usage evolution along the predominant mutational pathway. This time scale was measured by the estimated number of months between the start of the evolution of CXCR4 use (the midpoint of the branch on which the first mutations promoting CXCR4 usage were mapped) and the earliest CXCR4-using ancestor (the midpoint of the earliest branch with a predicted FPR below 3.5). These intervals ranged from 4.1 (DS7) to 30.6 (DS2) months with a mean of 16.6 months. To assess whether there was any association between these time intervals and the waiting time until the emergence of the first mutations promoting CXCR4 use, we calculated these quantities for all ancestral lineages in all subjects that eventually attained a predicted FPR below 3.5. We found a significant negative correlation between the waiting time and time scale of evolution (one-sided Pearson's 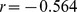, df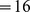, 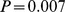) that is consistent with a trade-off between gradual and fitness-valley modes of evolution. However, this correlation is biased by the non-independent evolution of lineages within the same subject. If we adjust for this by averaging values across lineages per subject, the correlation remains negative but is no longer significant (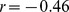, 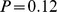).

**Figure 5:**
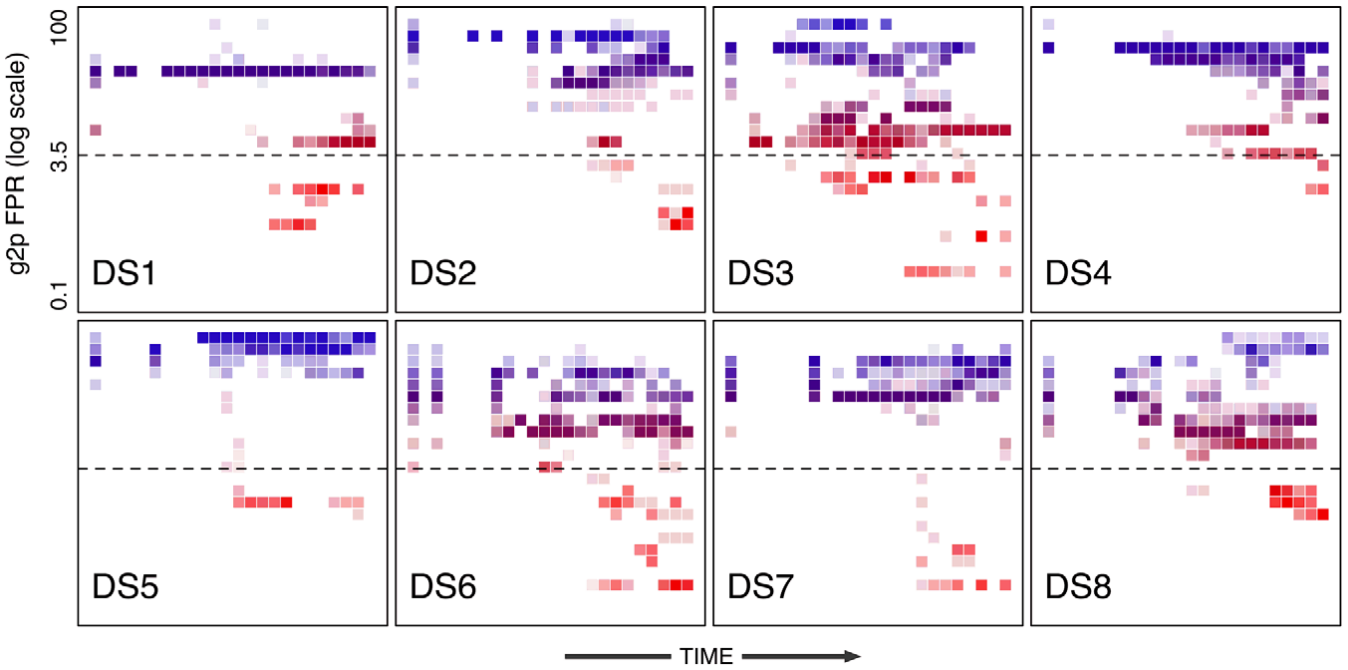
Two-dimensional histograms illustrating the distributions of g2p FPR predictions across all replicate ancestral reconstructions on the maximum credibility tree. The 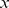-axis corresponds to time intervals from 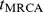 to the first positive MT-2 assay (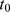), rescaled for each subject. The 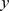-axis corresponds to the log-transformed FPR predictions for the ancestral sequences. Both axes were partitioned into 25 bins. Each cell is coloured with respect to its FPR value with opacity proportional to the square root of the number of data points in the corresponding bins, normalized by the total number of points in the time interval.

One of the drawbacks to the preceding visualization schemes is that they focus on the mutations or a subset of mutations comprising the MARCS. To summarize all replicate ancestral reconstructions on the maximum credibility tree for each subject, we generated 2-dimensional histograms summarizing the distributions of coreceptor usage predictions for all reconstructed ancestral sequences over time. Darker shaded cells represent higher densities in the corresponding intervals of reconstructed FPR predictions, normalized for a given time interval (Figure 5). In general, each distribution broadens over time (from left to right) as the accumulation of genetic variation manifests itself in the diversification of coreceptor usage predictions. The histograms derived from our analysis of sequence variation from subjects DS5 and DS7 are characterized by a distinct and rapid bifurcation into low FPR values from a main trunk of high FPR values (Figure 5). We have already determined that this dynamic in subject DS7 can be attributed to the rapid accumulation of mutations after a substantial delay (Figure 3) that is consistent with the fitness valley model of HIV coreceptor usage evolution. The map of mutations to the maximum credibility tree reconstructed from DS5 sequences is also consistent with this interpretation (Supporting Figure S5). However, the reconstructed dynamics in the remaining six subjects exhibited finer gradation in FPR values over time (Figure 5). An interesting feature of these histograms is the emergence of lineages (for example, in subjects DS3 and DS8) with a greater tendency for CCR5 usage than the MRCA, which represents the putative transmitted variant. Consequently, the predominant CCR5-using variant at the time of sampling is not necessarily representative of the CCR5-using ancestor from which CXCR4-using lineages are derived, which can only be revealed by ancestral reconstruction using phylogenetic methods.

## Discussion

Our findings indicate that a substantial fraction of the evolutionary history of HIV coreceptor usage preceded the first samples from these subjects by entire years, a significant amount on the time scale of HIV evolution. Consequently, the direct comparison of observed sequences, such as by a minimum-spanning tree [12], is insufficient to determine whether these data support the transmission-mutation hypothesis, which stipulates a fitness valley separating CCR5- and CXCR4-using genotypes [5]. We have shown how phylogenetic methods can be used to reconstruct the ancestral HIV sequences from which the observed data descend. The availability of samples from different points in time enabled us to estimate the rates of HIV evolution using molecular clock models. In turn, this enabled us to date branches in the phylogeny down to the most recent common ancestor, and to date the emergence of specific mutations. We found that the reconstructed evolutionary dynamics of HIV coreceptor usage did not equivocally support the fitness valley postulated by the transmission-mutation hypothesis, although there were two cases (DS5 and DS7) where dynamics were consistent with the presence of a fitness valley. Additionally, we found some evidence of a negative correlation between the time to the onset of the HIV coreceptor switch and the duration of the switch itself. Hence, the dynamics of the HIV coreceptor switch cannot be explained by a single model because it is dependent on the genotype of the transmitted variant, which determines the pathways available to evolve CXCR4 usage. In other words, these results suggest that not every HIV infection begins at the peak of a fitness valley with respect to HIV coreceptor usage. Furthermore, if the role of a fitness valley in shaping the evolution of HIV coreceptor usage is contingent on the genotype of the transmitted variant, as implied by our results, then the probability of the HIV coreceptor switch may be a heritable trait among transmitted virus lineages.

This study makes use of next-generation sequencing to automate the process of sequencing individual nucleic acids from the sample population (clonal sequencing), which is otherwise time-consuming and less scaleable. Specifically, we use an ‘ultra-deep’ application of next-generation sequencing, which generates thousands of reads from the same region of each nucleic acid to yield a large sample of genetic variation that is ideal for phylogenetic analysis. A potential hazard of this application is that the large number of reads may outnumber the actual number of HIV RNA copies from the specimen, leading to excessive resampling of genetic variation. We have estimated that sample processing would result in about a 20-fold dilution in the number of nucleic acids (see Methods section). While this is substantial, the serum samples most likely contained high numbers of nucleic acids because they were drawn from treatment naïve subjects with chronic HIV infections. The median viral load previously reported from these subjects was 17,000 HIV RNA copies/mL [12]. We would therefore estimate that about 850 nucleic acids would be represented by copies at the sequencing stage of the sample processing protocol. Given that we used only 50 random sequences from each sample, the probability of subsampling was about 0.2% per molecule.

Additionally, we directly measured the number of HIV RNA copies available for sequencing by using recently-developed ‘primer ID’ technique [38]. This technique employs a partially-degenerate primer in the reverse transcription reaction that causes each complementary DNA strand to be labelled by a random string of nucleotides. Consequently, sequences sharing the same primer ID will have been derived from the same nucleic acid at the initial phase of RT-PCR amplification. In other words, the number of unique primer IDs should correspond to the number of nucleic acids transferred from the extraction eluate to the RT-PCR reactions in triplicate. Because the original sera samples were too depleted, we reprocessed frozen HIV RNA extracts from four samples using a primer containing a degenerate 9-mer. These samples corresponded to two time points from DS8 at +3 and +6.8 months since the first positive MT-2 assay (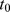), one from DS2 at +6 months since 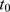, and one from DS6 at +3 months. Viral loads were previously reported to be approximately 1000 HIV RNA copies per mL at both DS8 time points [12]; no viral load measurements were available at the time points from either DS2 or DS6. The numbers of unique primer ID sequences in the resulting deep sequence data were 400, 317, 2003, and 343, respectively. Hence, there was a low probability (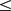
1%) that copies of the same nucleic acid were resampled in a random selection of 50 sequences per time point, even when the reported viral loads associated with these samples were relatively low.

Molecular clock models are an important application of phylogenetic reconstruction. We have taken advantage of serial samples with known dates to calibrate the molecular clock, which enables us to reconstruct the evolutionary history of the HIV populations back in time [18]. For example, we extrapolated the time scale of HIV coreceptor usage evolution back to most recent common ancestors, which we estimated to have preceded the times of the first positive MT-2 assay by 2 to 5 years. There are some caveats to be aware of when interpreting estimates of 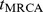 from a molecular clock analysis. First, a strong selective sweep could conceivably replace the MRCA with a more recent ancestor. Published estimates of 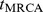 from individuals with known or estimated times of transmission, however, tend to be consistent with, or moderately overestimate, those times [29], [39]. In addition, the high rate of recombination in HIV-1 can limit the effect of a selective sweep to a narrower interval of the genome, although it may also raise other issues related to phylogenetic reconstruction (see below). Second, the nucleotide substitution models typically used for molecular clock analyses may become saturated for highly divergent lineages, causing one to underestimate the actual 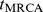, although this effect has only been reported for the large-scale divergence of virus populations among hosts (for example, dating the zoonotic origin of measles virus from rinderpest virus [40]). If saturation was present within hosts, it will have been ameliorated by our use of separate model parameters for the third codon position that is more susceptible to this effect [28], [40].

Recombination can result in phylogenetic incongruence, in which different regions of a genome are related by different phylogenies. Although the sequences analyzed here were relatively short (about 250 bp), we cannot rule out that within-host recombination within this interval may have interfered with accurate reconstruction of the phylogeny or ancestral sequences. For example, multiple lineages in the phylogeny reconstructed for subject DS8 accumulated the same mutations within the V3 loop (S11G, D25K, Q10R and H13R; see Supporting Figure S5) that could conceivably have been transferred from the same parent lineage into different genomic backgrounds. However, this putative case of parallel evolution cannot be readily explained by recombination because many observed sequences derived from these lineages contained only intermediate subsets of these mutations, which would have required multiple recombination events at consistent breakpoints between the same lineages in a relatively short period of time.

We used a Bayesian Markov chain Monte Carlo (MCMC) sampling procedure implemented in BEAST [26] because this is currently the best-maintained software for fitting a molecular clock phylogeny to serial samples of genetic sequence data. One of the disadvantages of this procedure, however, is that the analysis becomes unfeasible when the total number of sequences substantially exceeds 200. This problem arises because the space of all possible trees becomes too large for an MCMC sampler to converge to the posterior distribution in a realistic amount of time. The use of serial samples can ameliorate this limit to some extent because it can constrain the range of trees to explore. We addressed this problem by limiting our analysis to only 50 randomly subsampled sequences per time-point. The resulting sample size should have been sufficient to characterize the principal trends in the dynamics of HIV evolution within these subjects; this is supported by the reproducibility of our results using a second set of random subsamples (Supporting Figure S2). Nevertheless, this is clearly a small fraction of the number of reads produced by next-generation sequencing; indeed, it is closer to the numbers yielded by conventional clonal sequencing. Thus, there remain considerable computational challenges to making full use of next-generation sequencing data from rapidly-evolving virus populations. For example, this problem may be amenable to recent innovations in sequential Monte Carlo methods, although development in this area is at an early stage [41].

It is important to note that our analysis was performed on samples from a retrospective study where the HIV infections were determined to have undergone a coreceptor switch by both phenotypic and genotypic assays. This pre-existing study criterion prevents us from drawing conclusions on the genetic determinants of whether an HIV infection will undergo a coreceptor switch. Further investigations will require a larger sample size including longitudinal samples from subjects without any positive SI or CXCR4-usage phenotype assay to identify the genetic determinants in the transmitted variant of the incidence and subsequent dynamics of the HIV coreceptor switch. Testing these hypotheses will require ‘time-stamped’ phylogenetic methods of ancestral reconstruction, including the analytical and visualization techniques we have developed in this study.

## Supporting Information

Figure S1 Comparison of a minimum spanning tree and phylogeny. Open circles represent observed sequences. A minimum spanning tree (red dashed lines) makes connections between these observations as a graphical representation of similarity. Shaded circles represent latent (ancestral) sequences that cannot be observed and must instead be inferred from the observed data. A phylogeny (solid lines) makes connections between observed and ancestral sequences that are inferred under a model of sequence evolution.
(PDF)

Figure S2 Two-dimensional histograms illustrating the distributions of g2p FPR predictions across all replicate ancestral reconstructions on the maximum credibility tree (see Figure 5). These histograms were generated from a second data set comprising new random samples of 50 sequences from each time point.
(PDF)

Figure S3 Posterior traces from replicate chain samples from a Bayesian MCMC-based molecular clock analysis of longitudinal HIV sequence datasets from eight subjects. The Gelman-Rubin convergence diagnostic (GD) point estimate is reported in the lower-left of each plot.
(PDF)

Figure S4 Schematic diagram of the bioinformatic workflow. The filenames of scripts written in Python or HyPhy batch language (unless otherwise indicated) are displayed in the lower half of each node.
(PDF)

Figure S5 Evolution of HIV coreceptor usage mapped to maximum credibility trees for eight subjects. Branches in each tree are coloured with respect to the false positive rate (FPR) prediction derived from the g2p algorithm. A lower FPR value indicates greater confidence that the reconstructed ancestral genotype yielded a CXCR4-using virus. Amino acid substitutions (labelled by ancestral residue, position in the V3 loop, and derived residue) inferred from ancestral reconstructions are mapped to the corresponding branches of each tree. Annotated excerpts from the trees for DS2 and DS7 are presented in Figure 3.
(PDF)

Text S1 Estimating the efficiency of RNA extraction.
(PDF)

Text S2 Indel error correction algorithm based on pairwise codon sequence alignment.
(PDF)

Text S3 Algorithm for binary encoding of indel polymorphisms in a codon sequence alignment.
(PDF)
