# Supplementary figures and images for: Reconstructing the Dynamics of HIV Evolution within Hosts from Serial Deep Sequence Data
Source: PLoS Comput Biol. 2012 Nov 1;8(11):e1002753. doi: 10.1371/journal.pcbi.1002753 (PMC3486858; doi:10.1371/journal.pcbi.1002753)

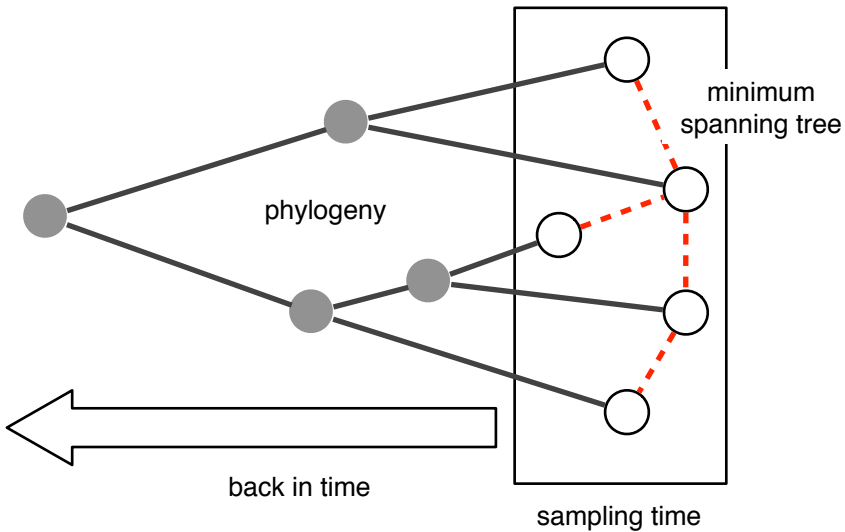

Supplement: Figure S1 — Comparison of a minimum spanning tree and phylogeny. Open circles represent observed sequences. A minimum spanning tree (red dashed lines) makes connections between these observations as a graphical representation of similarity. Shaded circles represent latent (ancestral) sequences that cannot be observed and must instead be inferred from the observed data. A phylogeny (solid lines) makes connections between observed and ancestral sequences that are inferred under a model of sequence evolution. (PDF) [file pcbi.1002753.s001.pdf]

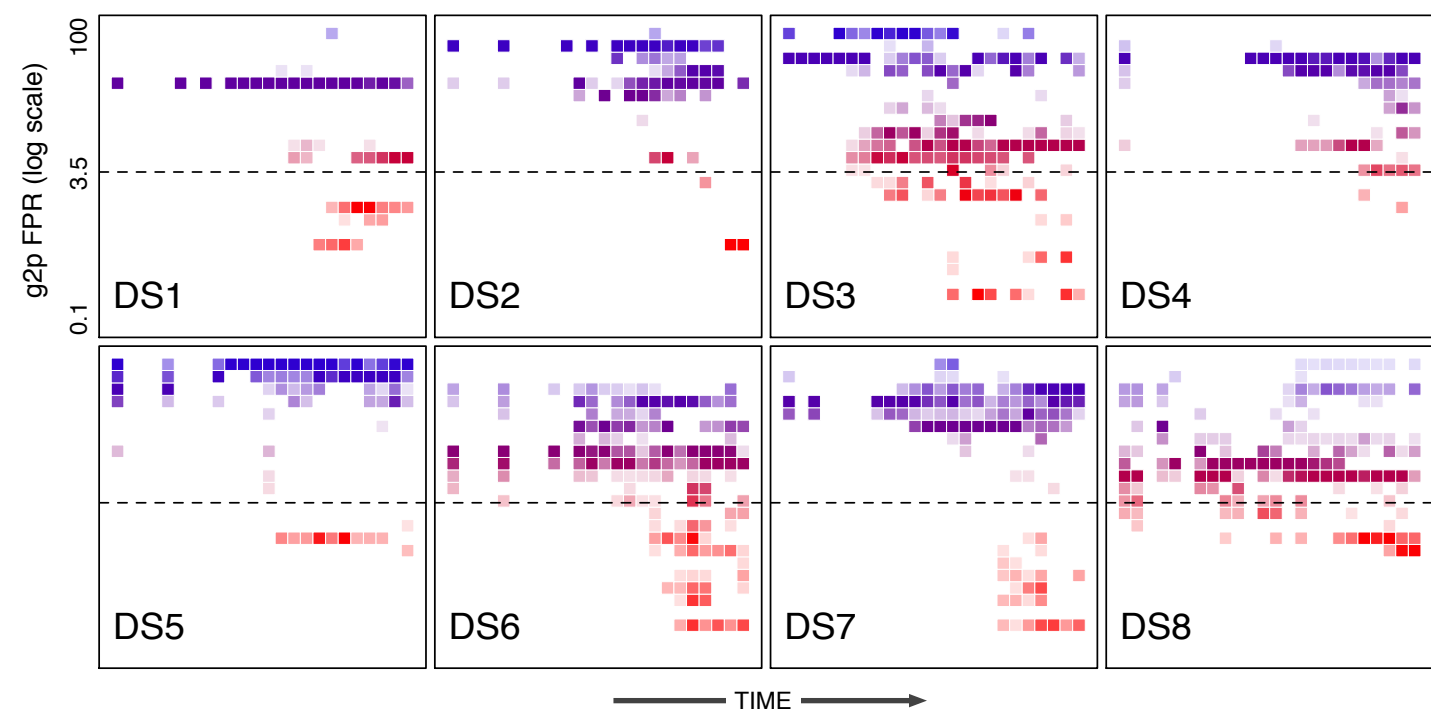

Supplement: Figure S2 — Two-dimensional histograms illustrating the distributions of g2p FPR predictions across all replicate ancestral reconstructions on the maximum credibility tree (see Figure 5). These histograms were generated from a second data set comprising new random samples of 50 sequences from each time point. (PDF) [file pcbi.1002753.s002.pdf]

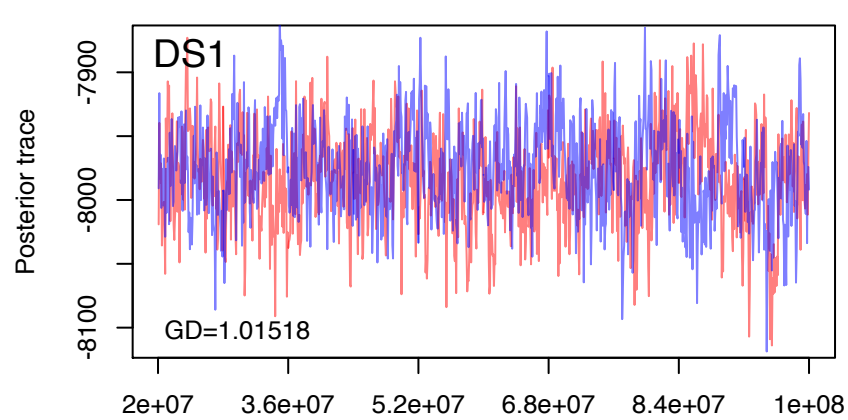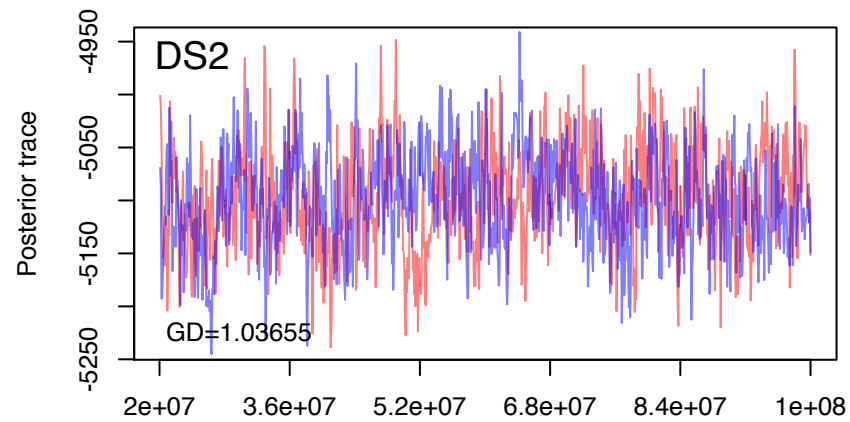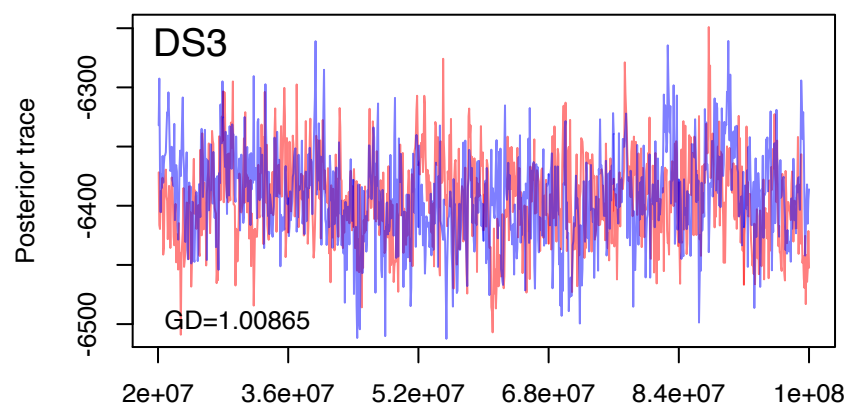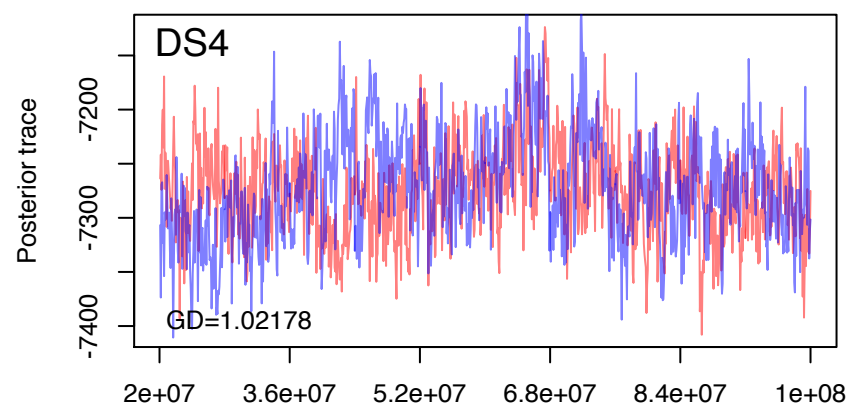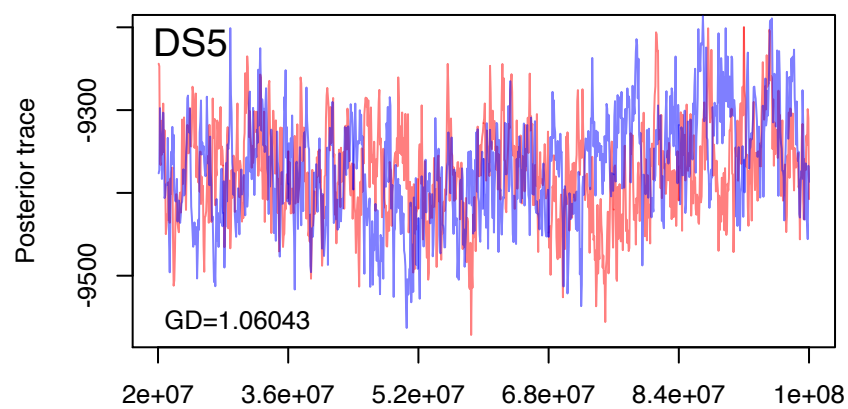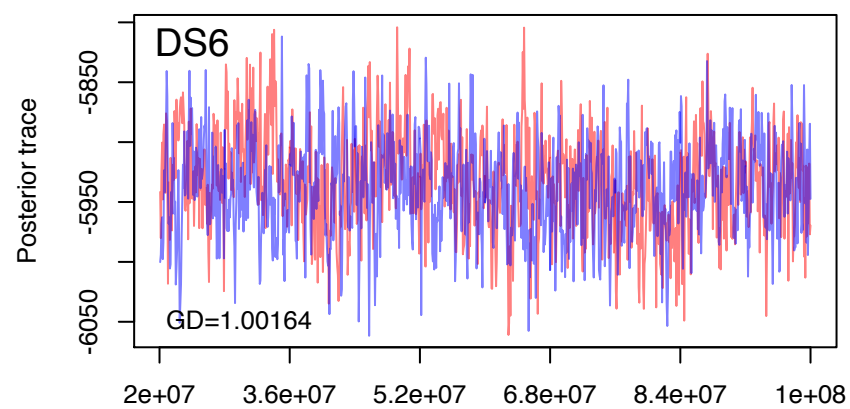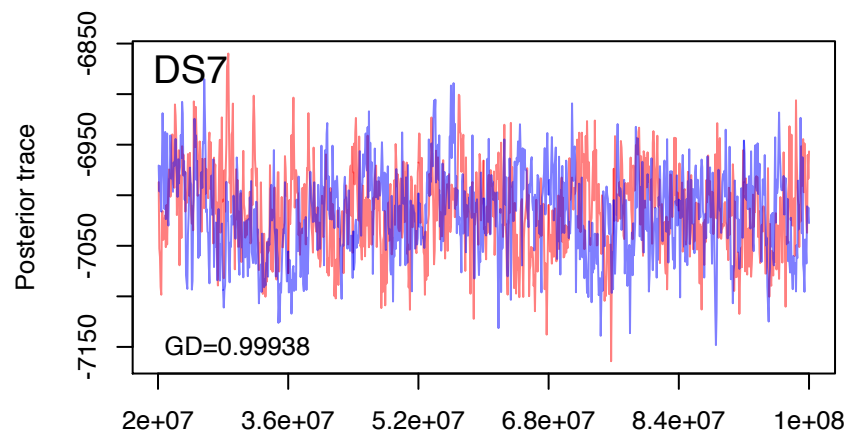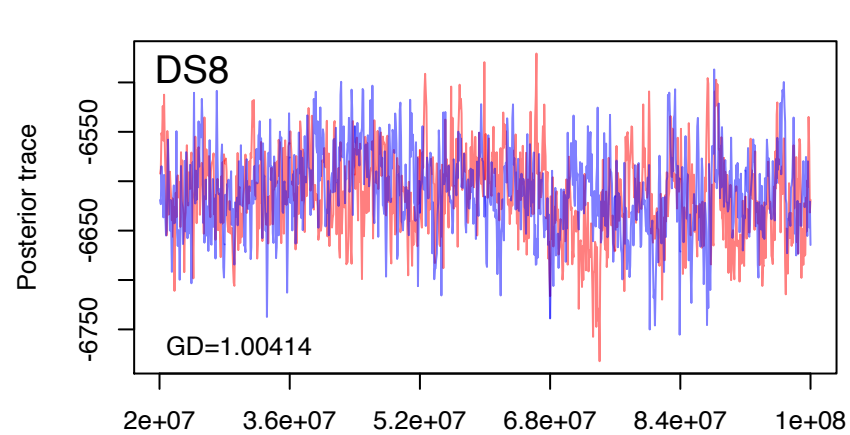

Supplement: Figure S3 — Posterior traces from replicate chain samples from a Bayesian MCMC-based molecular clock analysis of longitudinal HIV sequence datasets from eight subjects. The Gelman-Rubin convergence diagnostic (GD) point estimate is reported in the lower-left of each plot. (PDF) [file pcbi.1002753.s003.pdf]

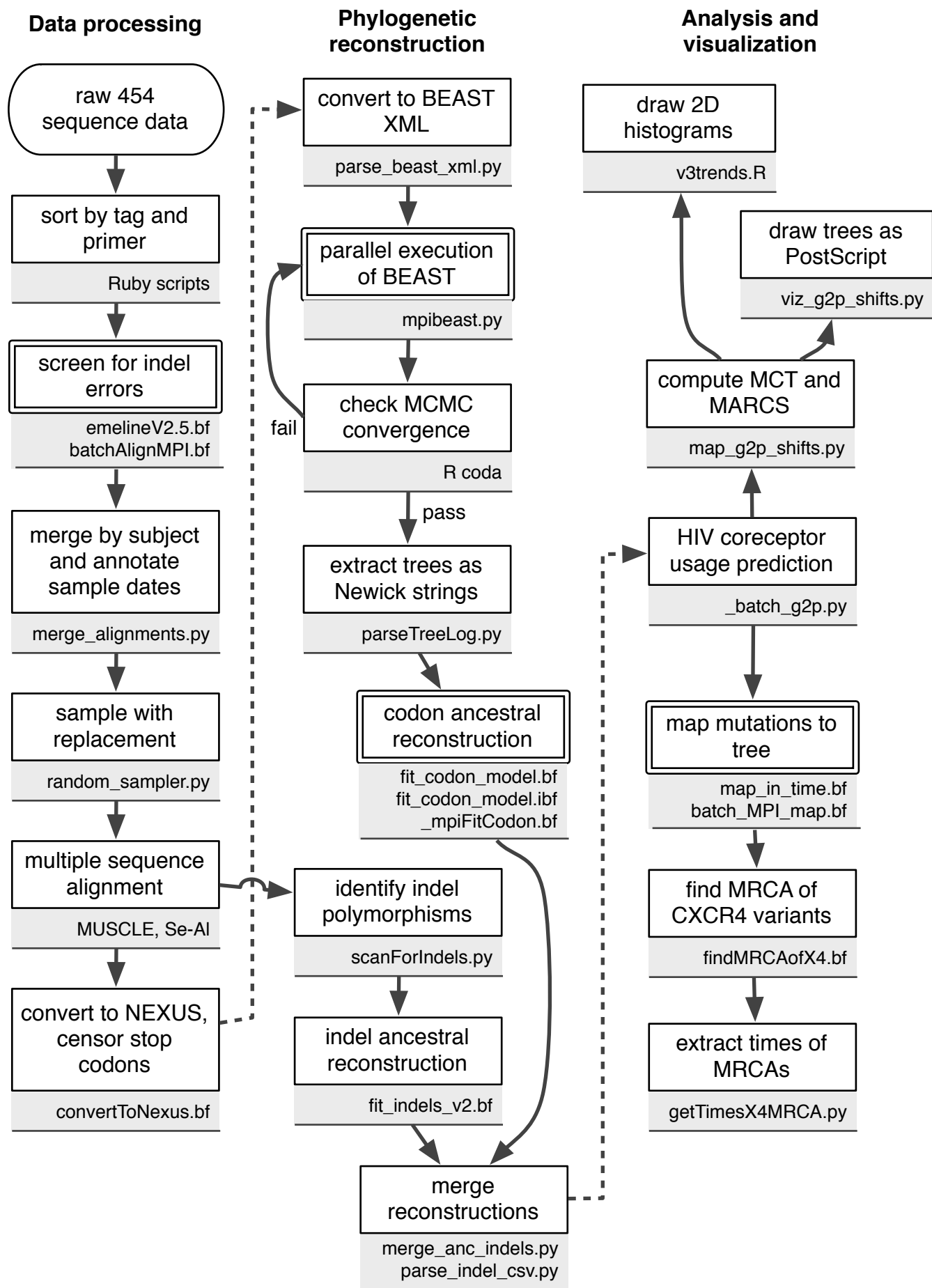

Supplement: Figure S4 — Schematic diagram of the bioinformatic workflow. The filenames of scripts written in Python or HyPhy batch language (unless otherwise indicated) are displayed in the lower half of each node. (PDF) [file pcbi.1002753.s004.pdf]

DS1

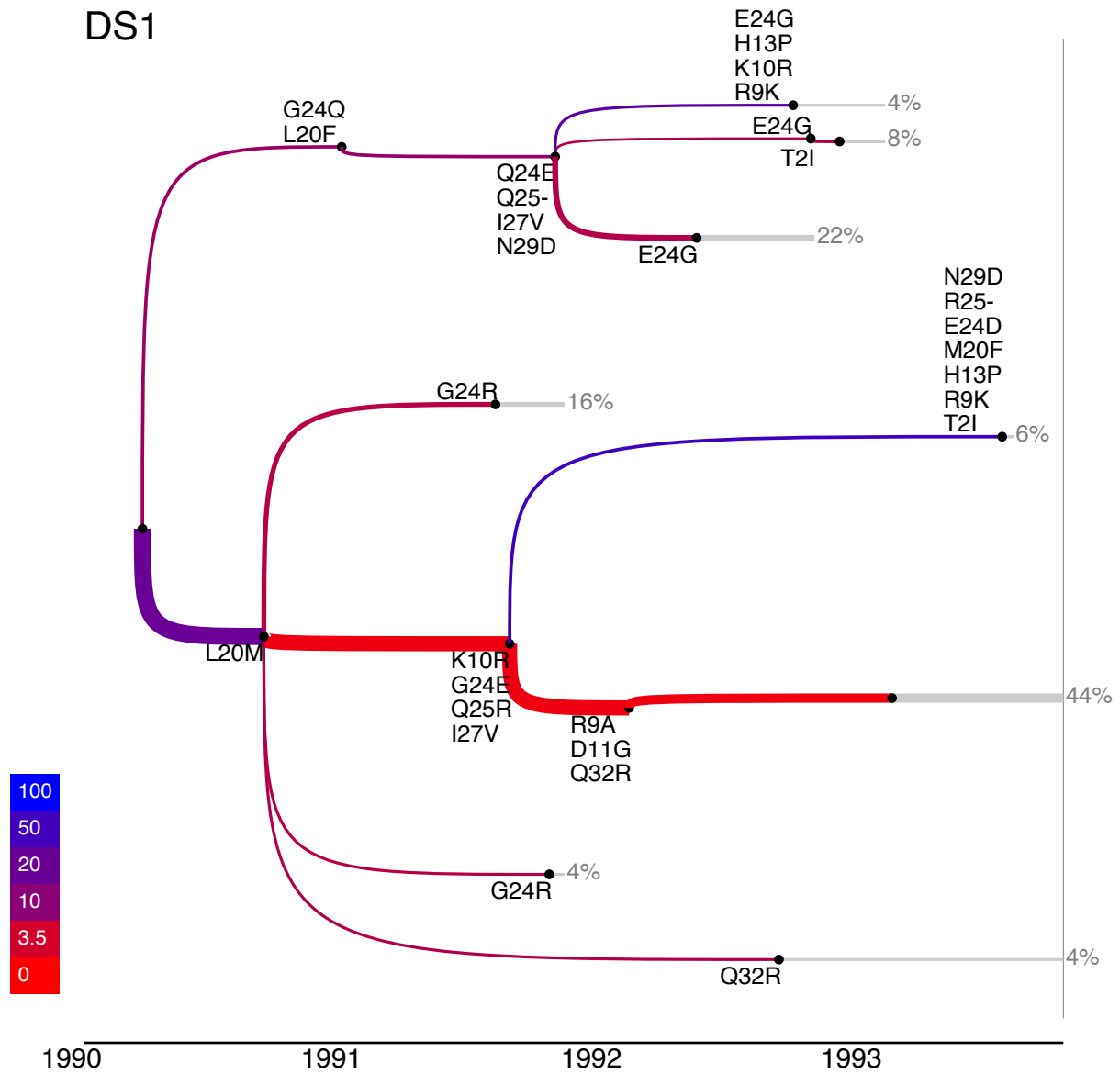

DS2

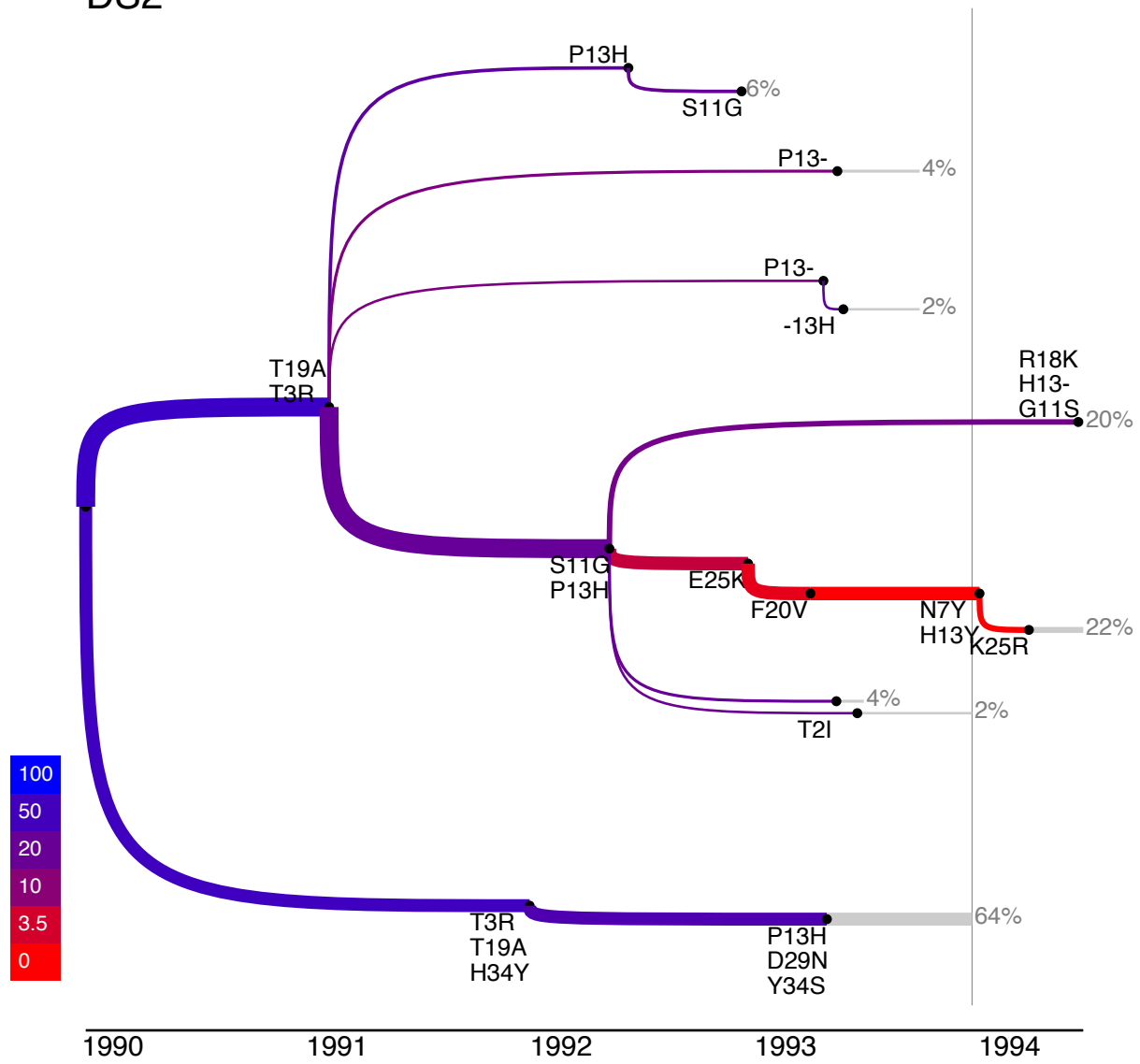

DS3

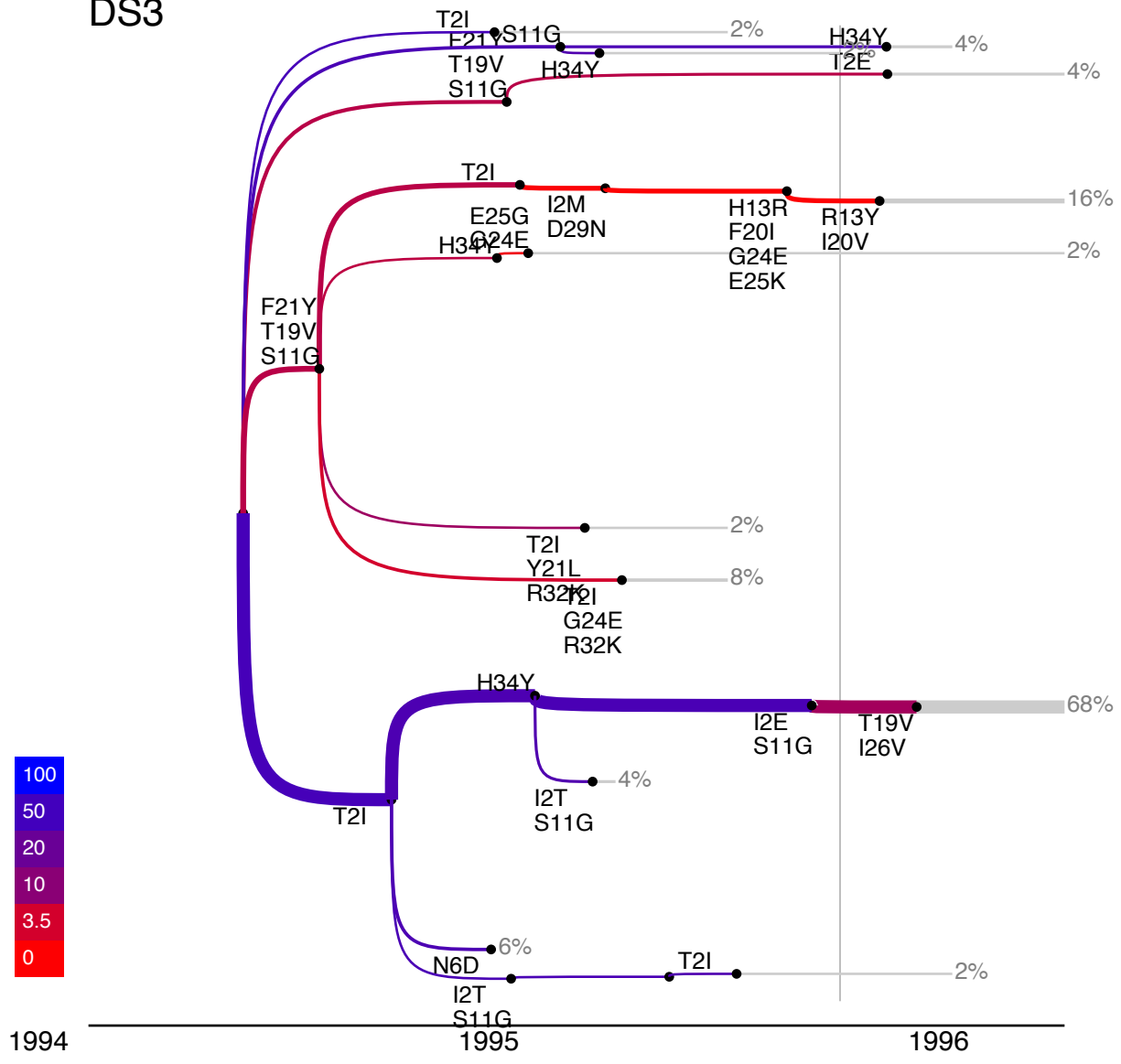

DS4

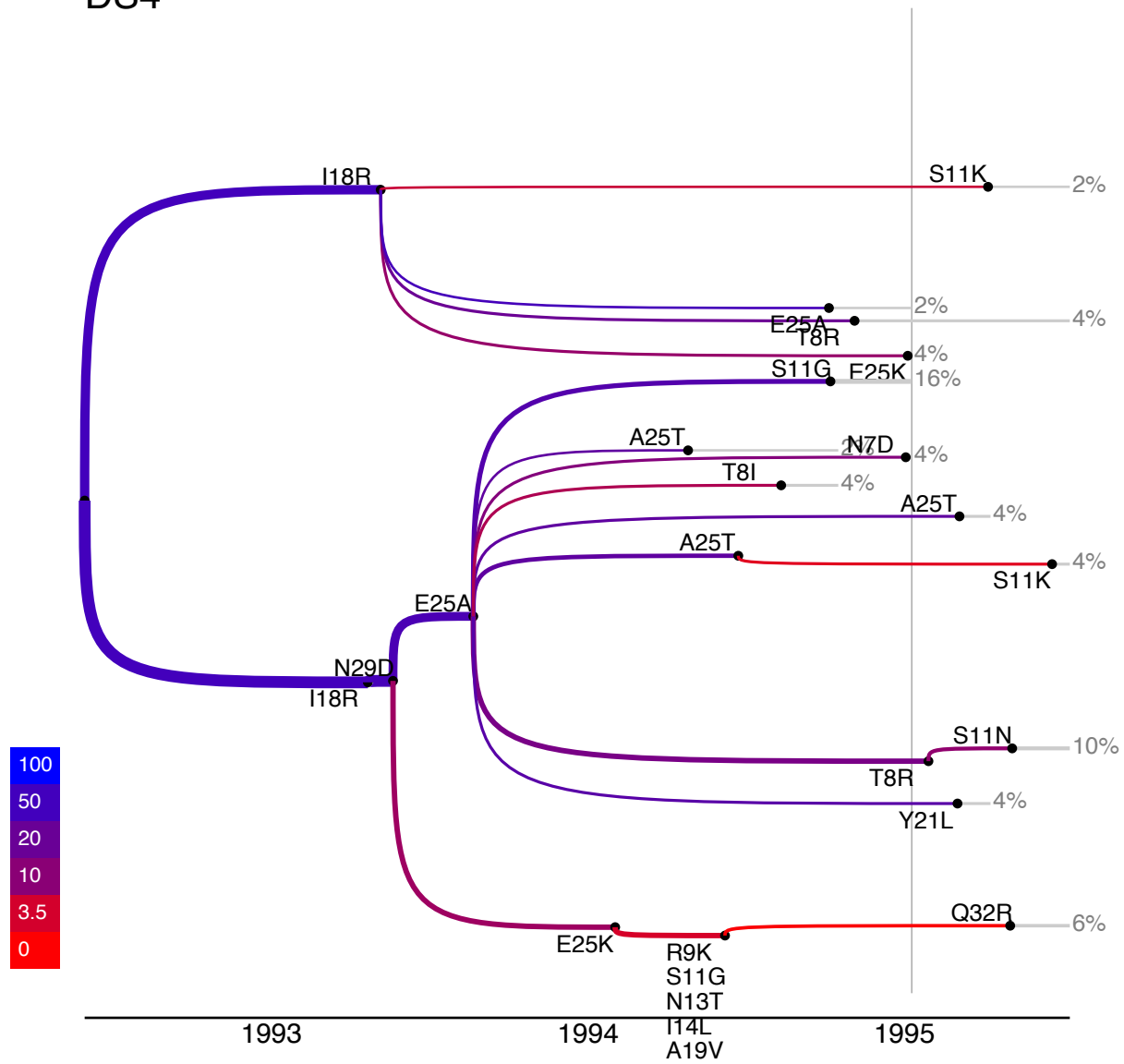

DS5

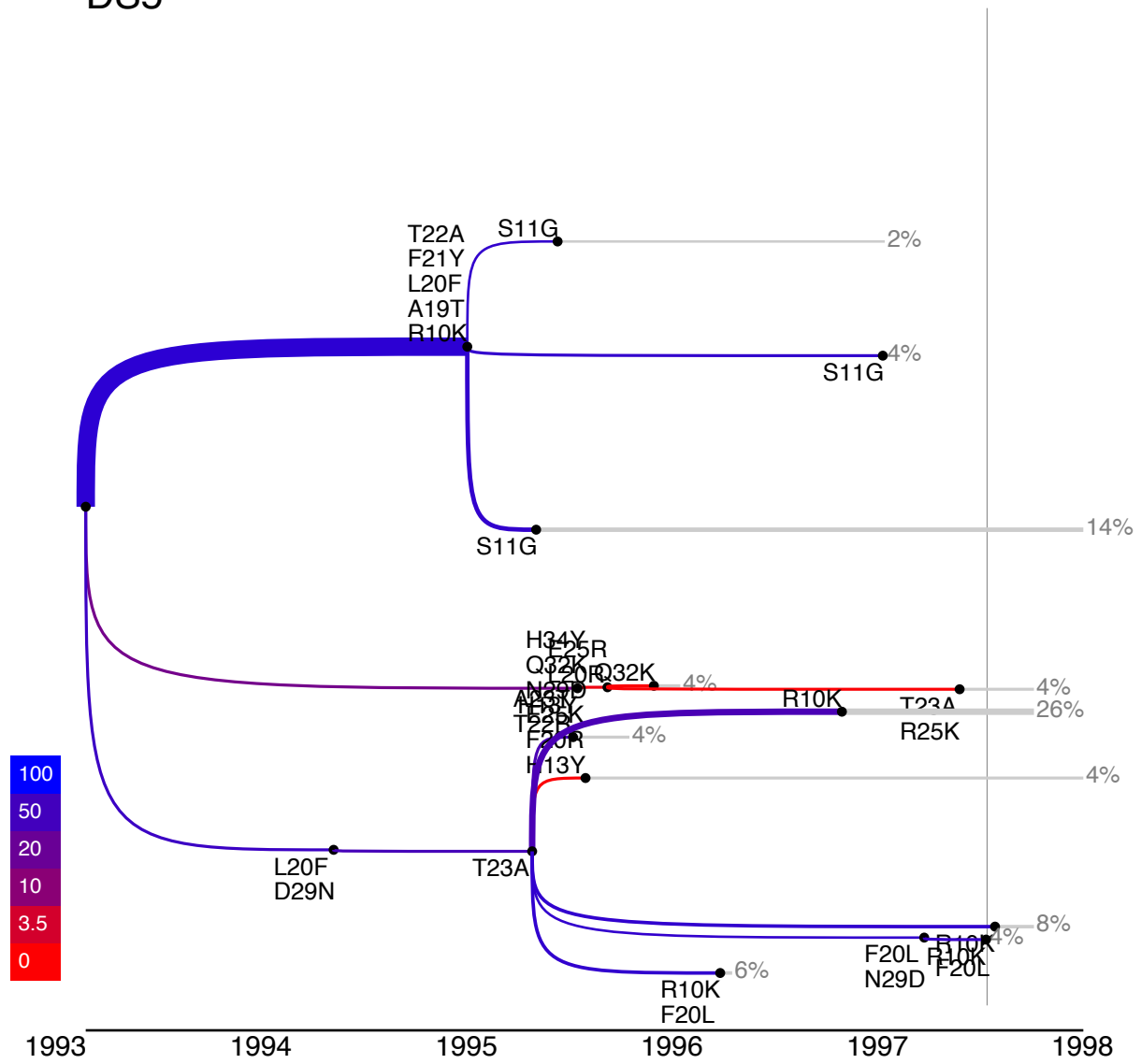

DS6

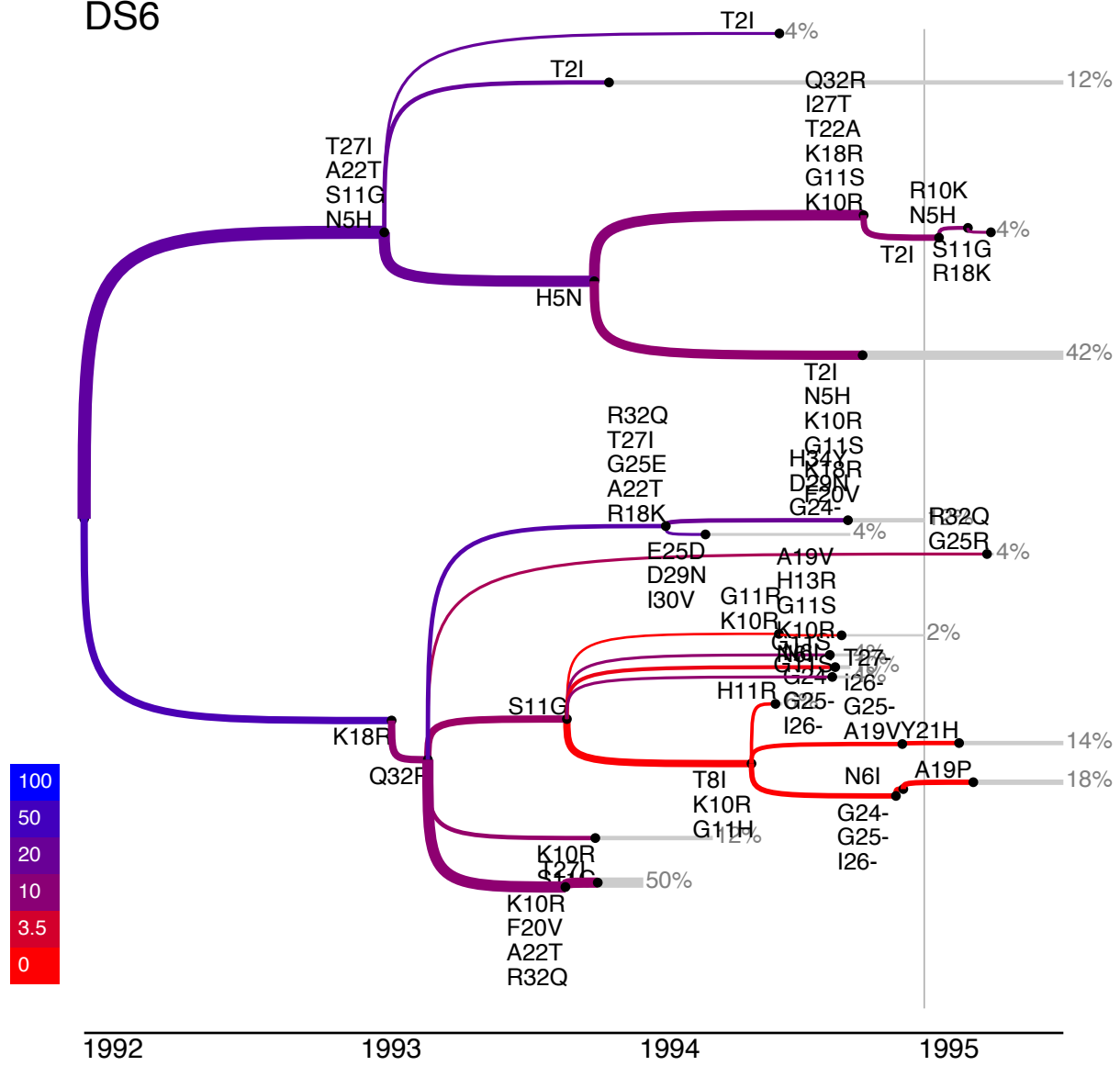

DS7

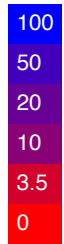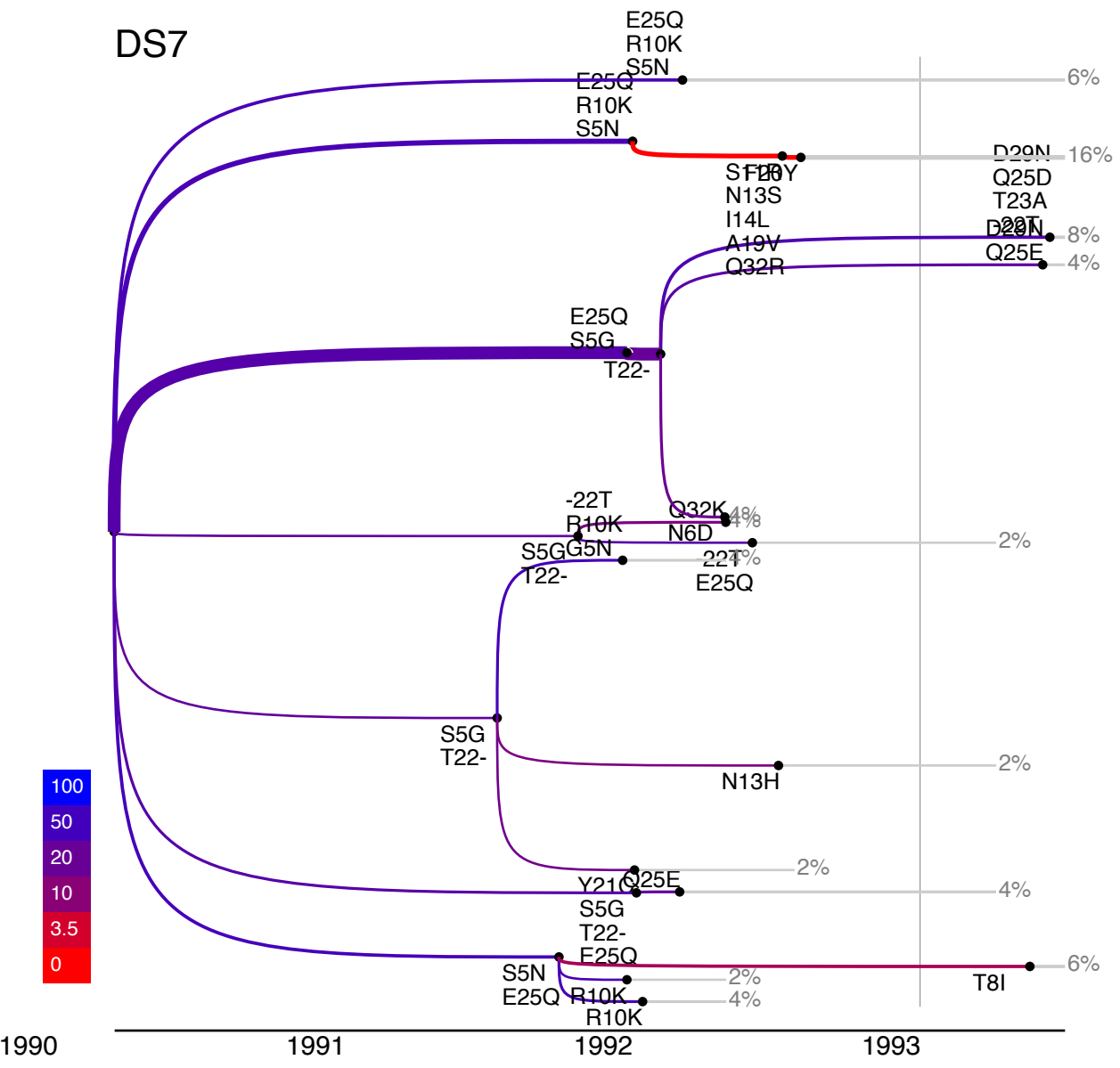

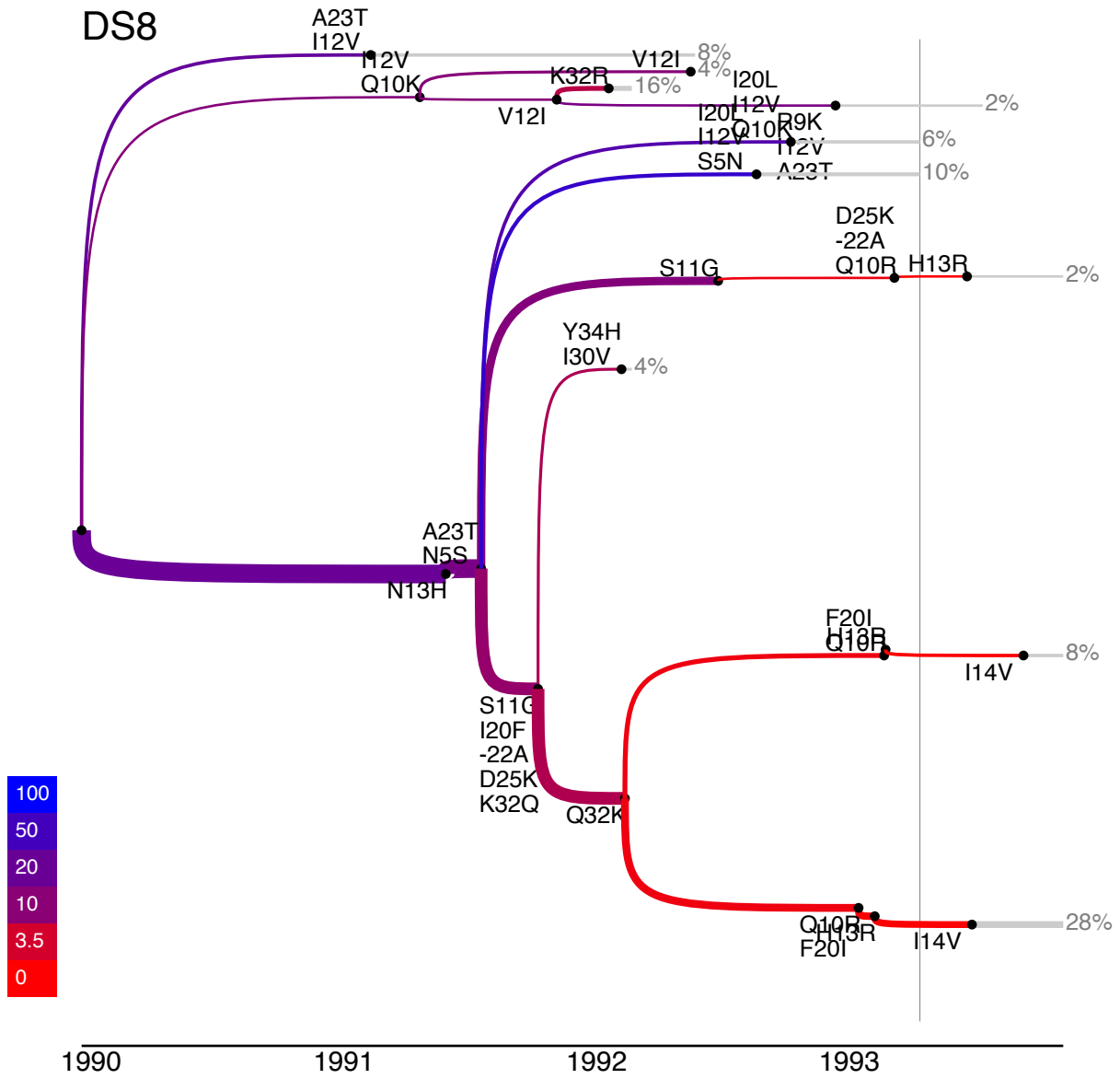

Supplement: Figure S5 — Evolution of HIV coreceptor usage mapped to maximum credibility trees for eight subjects. Branches in each tree are coloured with respect to the false positive rate (FPR) prediction derived from the g2p algorithm. A lower FPR value indicates greater confidence that the reconstructed ancestral genotype yielded a CXCR4-using virus. Amino acid substitutions (labelled by ancestral residue, position in the V3 loop, and derived residue) inferred from ancestral reconstructions are mapped to the corresponding branches of each tree. Annotated excerpts from the trees for DS2 and DS7 are presented in Figure 3. (PDF) [file pcbi.1002753.s005.pdf]
