# Supplementary material for: Reconstructing the Dynamics of HIV Evolution within Hosts from Serial Deep Sequence Data
Source: PLoS Comput Biol. 2012 Nov 1;8(11):e1002753. doi: 10.1371/journal.pcbi.1002753 (PMC3486858; doi:10.1371/journal.pcbi.1002753)
Supplement: Text S1 — Estimating the efficiency of RNA extraction. (PDF) [file pcbi.1002753.s006.pdf]

Supporting Text S1

The efficiency of RNA extraction using the NucliSENS easyMAG was estimated from published respiratory syncytical detection RNA detection results [1] where an estimated 5, 10, 25 and 50 copies of viral RNA from a dilution series were added to lysis buffer prior to extraction, respectively yielding 0, 3, 8 and 8 successful amplifications out of 8 replicates. Since only 20% of the extracted RNA was transferred to amplification reactions in that study, the likelihood of extraction efficiency is given by:

$$L(r) = Bn(0, 8, 1 - e^{r}) \times Bn(7, 8, 1 - e^{2r}) \times Bn(7, 8, 1 - e^{5r}) \times Bn(8, 8, 1 - e^{10r})$$

where $Bn(n, k, p)$ is the binomial distribution for n trials and k successes with probability p, and $1 - e^{-\lambda}$ is the Poisson probability of a non-zero count given rate λ. $L(r)$ is maximized at r = 0.444.

[1] Manji R, Lotlikar M, Zhang F, Ginocchio CC (2009) Clinical evaluation of NucliSENS magnetic extraction and NucliSENS analytical specific reagents for the real-time detection of respiratory syncytial virus (RSV) in paediatric respiratory specimens. J Clin Pathol 62: 998-1002.
