# Supplementary material for: Reconstructing the Dynamics of HIV Evolution within Hosts from Serial Deep Sequence Data
Source: PLoS Comput Biol. 2012 Nov 1;8(11):e1002753. doi: 10.1371/journal.pcbi.1002753 (PMC3486858; doi:10.1371/journal.pcbi.1002753)
Supplement: Text S2 — Indel error correction algorithm based on pairwise codon sequence alignment. (PDF) [file pcbi.1002753.s007.pdf]

Supporting Text S2

We assumed that HIV coding sequences maintained a single reading frame across the region being sequenced. Consequently, any shifts in reading frame could be attributed to indel errors introduced by next-generation sequencing. Based on this premise, we use the following algorithm to remove indel errors:

1. Each ‘query’ nucleotide sequence is aligned pairwise against the reference using a modified Smith-Waterman algorithm [1] without penalizing trailing gaps, and clipped to the interval that overlaps the reference sequence. This step also identifies the sequence direction relative to the reference – in other words, whether to use the reverse-complement of the query.
2. The reference sequence is clipped to the interval overlapping the query sequence with the highest alignment score. This maximizes the efficiency of subsequent steps.
3. Each query sequence is aligned against the reference protein sequence to determine the best overall reading frame.

For each query sequence, the algorithm attempts to find the path through all three reading frames that maximizes the alignment score to the reference protein sequence. Protein alignment is carried out using the modified Smith-Waterman algorithm with an empirical HIV amino acid score matrix [2] and penalties for trailing gaps. This is accomplished by the following recursive algorithm:

- Evaluate all possible frame-shifts along the query sequence. The search is constrained so that the substring to either the left or right of the frameshift retains the current ‘best’ reading frame.
- If the frame-shift improves the alignment score relative to the reference protein sequence, then split the sequence at the frame-shift and repeat the previous step for each substring.
- A recursion exits when there is no improvement of alignment score with the addition of any frame-shift, or at a maximum recursion depth of three.

Results from the preceding step are used to generate a new nucleotide sequence with gaps inserted to compensate for frame-shift inducing deletions. Insertions that induce frame-shifts are stripped from the sequence. As a result, a single reading frame is enforced along the entire length of the new sequence.

[1] Gotoh O (1982) An improved algorithm for matching biological sequences. J Mol Biol 162: 705-8.

[2] Nickle DC, Heath L, Jensen MA, Gilbert PB, Mullins JI, et al. (2007) HIV-specific probabilistic models of protein evolution. PLoS One 2: e503.
