# Supplementary material for: Reconstructing the Dynamics of HIV Evolution within Hosts from Serial Deep Sequence Data
Source: PLoS Comput Biol. 2012 Nov 1;8(11):e1002753. doi: 10.1371/journal.pcbi.1002753 (PMC3486858; doi:10.1371/journal.pcbi.1002753)
Supplement: Text S3 — Algorithm for binary encoding of indel polymorphisms in a codon sequence alignment. (PDF) [file pcbi.1002753.s008.pdf]

Supporting Text S3

An indel polymorphism was defined as a continguous interval in the codon sequence alignment containing one or more codon gaps in any sequence. The polymorphism may be comprised of two or more character states corresponding to the respective lengths and/or locations of each codon insertion or deletion. We encoded these character states as integer values in decreasing order of prevalence, such that (0) represented the most common character state. For example, consider following alignment of codon sequences:

```
GGAGGAATAACAGGA
GGA------ACAGGA
GGAGGA------GGA
```

Relative to the topmost sequence, we find two deletions of the same length (2 codon gaps) and different locations. By treating these deletions as alternate character states at the same indel polymorphism, we are making the assumption that they are mutually-exclusive outcomes of sequence evolution. Alternatively, one could handle this scenario as three independently-evolving indel polymorphisms comprising single codon deletions each. This approach is less parsimonious, however, and it would imply that all possible combinations of these polymorphisms could be observed or reconstructed in the ancestral sequences. We use the following algorithm to encode indel polymorphisms from a codon sequence alignment:

1. Encode the presence (1) or absence (0) of gap characters for each codon in the sequence as a binary sequence.
2. Locate the shortest contiguous non-overlapping intervals in the binary sequences. Each interval is subsequently handled as a distinct indel polymorphism.
3. Collapse each binary sequence into an integer-valued sequence where each integer corresponds to an indel polymorphism as defined above, numbered in order of decreasing prevalence.

To illustrate, consider the expanded example:

Sequence	Count	Binary	Encoding
GGAGGAATAACAGGACATTTT---AAT	127	000000010	00
GGA------ACAGGACATTTTAAGAAT	8	011000000	11
GGAGGA------GGACATTTTAAGAAT	1	001100000	21

where the codon sequence intervals that became interpreted as indel polymorphisms have been underlined.
